# Inhibition of Rho-associated kinases disturbs the collective cell migration of stratified TE-10 cells

**DOI:** 10.1186/s40659-015-0039-2

**Published:** 2015-09-02

**Authors:** Taro Mikami, Keiichiro Yoshida, Hajime Sawada, Michiyo Esaki, Kazunori Yasumura, Michio Ono

**Affiliations:** Department of Histology and Cell Biology, Yokohama City University School of Medicine, Yokohama, Kanagawa-ken, Japan; Department of Plastic and Reconstructive Surgery, Fujisawa Shounandai Hospital, Fujisawa, Kanagawa-ken Japan; Department of Plastic and Reconstructive Surgery, Yokohama City University Hospital, Yokohama, Kanagawa-ken Japan

**Keywords:** Collective cell migration, Stratified epithelium, TE-10 cells, Rho-associated coiled coil-containing protein kinase, siRNA

## Abstract

**Background:**

The collective cell migration of stratified epithelial cells is considered to be an important phenomenon in wound healing, development, and cancer invasion; however, little is known about the mechanisms involved. Furthermore, whereas Rho family proteins, including RhoA, play important roles in cell migration, the exact role of Rho-associated coiled coil-containing protein kinases (ROCKs) in cell migration is controversial and might be cell-type dependent. Here, we report the development of a novel modified scratch assay that was used to observe the collective cell migration of stratified TE-10 cells derived from a human esophageal cancer specimen.

**Results:**

Desmosomes were found between the TE-10 cells and microvilli of the surface of the cell sheet. The leading edge of cells in the cell sheet formed a simple layer and moved forward regularly; these rows were followed by the stratified epithelium. ROCK inhibitors and ROCK small interfering RNAs (siRNAs) disturbed not only the collective migration of the leading edge of this cell sheet, but also the stratified layer in the rear. In contrast, RhoA siRNA treatment resulted in more rapid migration of the leading rows and disturbed movement of the stratified portion.

**Conclusions:**

The data presented in this study suggest that ROCKs play an important role in mediating the collective migration of TE-10 cell sheets. In addition, differences between the effects of siRNAs targeting either RhoA or ROCKs suggested that distinct mechanisms regulate the collective cell migration in the simple epithelium of the wound edge versus the stratified layer of the epithelium.

**Electronic supplementary material:**

The online version of this article (doi:10.1186/s40659-015-0039-2) contains supplementary material, which is available to authorized users.

## Background

The collective migration of epithelial cells is an important phenomenon in wound healing [1, 2], development [3–5], and cancer invasion [6–8]. The migration of stratified epithelial cells has been observed in wound healing that occurs in the skin, esophagus, vagina, and other organs. However, it is generally difficult to observe the collective migration of live epithelial cells in vivo because this type of cell migration generally occurs under scabs, eschars, or within the body [9]. Furthermore, although collective cell migration of the simple epithelium has been well investigated in vitro [10, 11], little is known in this regard of the stratified epithelium perhaps, in part, because of the difficulties inherent in such studies on this tissue. Therefore, to study the collective cell migration of stratified epithelium in vitro, novel methods are required to induce the formation of stratified epithelium from epithelial cells and to visualize their collective migration [12, 13].

Rho family proteins, which comprise small GTPases such as RhoA [14, 15], Rac1 [11, 16], and Cdc42 [17], play important roles in cell migration by mediating the organization of various types of actin filaments [18]. RhoA induces the formation of stress fibers by activating Rho-associated coiled coil-containing protein kinases (ROCKs). ROCKs phosphorylate many kinds of substrates such as myosin light chain (MLC) [19], Lin-11 Isl-1 Mec-3 kinase (LIMK) [20], MLC phosphatase (MYPT) [21], and tubulin polymerization promoting protein (TPPP1/p25) [22], all of which are related to cell migration [23]. Pharmacological inhibition of ROCKs has been shown to inhibit the migration of some cell types [24–27] while promoting the migration of others [28, 29]. Thus, the exact role of ROCKs in cell migration is controversial and might be cell-type dependent. In addition, the effects of pharmacological inhibition of ROCKs on the migration of specific cell lines derived from renal tubular epithelium have been shown to depend on the type of extracellular matrix to which the cells adhered [30], indicating that ROCK inhibition exerts differential as well as cell type-dependent effects on cell migration. Furthermore, in a study using clinical samples collected from patients with esophageal cancer, ROCK inhibition suppressed the invasion and metastasis of esophageal cancer cells [31], suggesting the importance of ROCKs in esophageal cancer progression.

Here, we describe a novel method to observe the collective migration of stratified cells on glass coverslips. Using this method to investigate the effects of Rho family proteins on collective cell migration, we found that the inhibition of ROCKs suppressed the collective migration of TE-10 cells, a cell line derived from human esophageal cancer tissue.

## Results

### High-density cultures

**Fig. 1:**
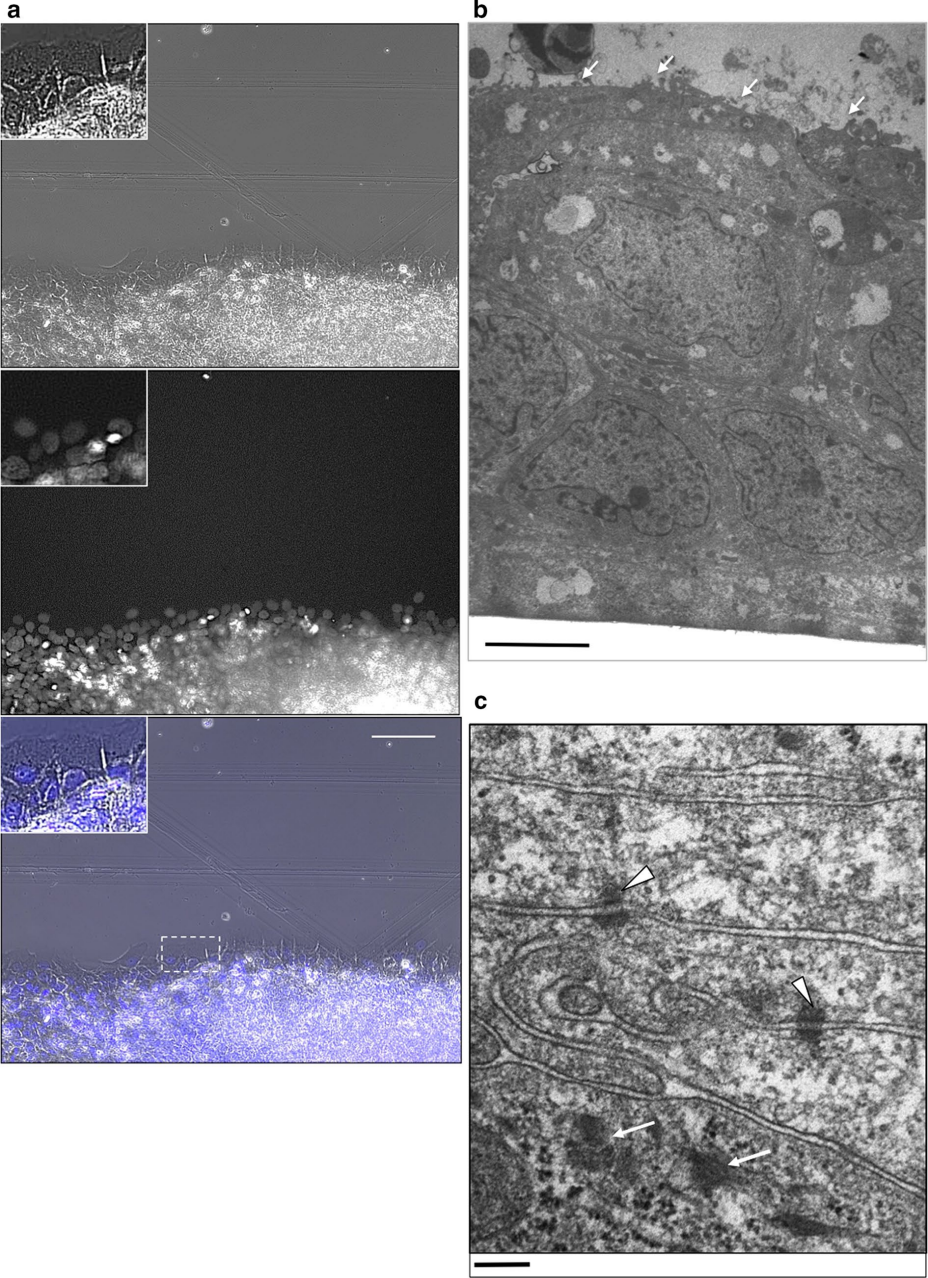
Images of stratified TE-10 cells in the wound edge and cross sections. **a** A randomly selected area of the wound edge was observed 24 h after scrape-induced wounding. Cells in the third row and deeper were multilayered, as indicated by the presence of overlapping nuclei. The *top panel* shows a phase-contrast image, while a nuclear-stained fluorescent image is presented in the *middle panel*. A merged image is shown in the *lower panel*. Each *inset* is the magnified image of the portion indicated in the merged image. The *scale bar* indicates 100 µm. **b** Transmission electron micrographs of the cross sections of stratified TE-10 cells plated in a hole of a silicone stencil at high density for 24 h. The cells were stratified into 5–7 layers from the apical to the basal side (the *upper region* of the image), which was attached to the glass cover slip. Few morphological differences were observed between the apical and basal sides, with the exception of microvillus formation (*white arrows*) at the surface of most apical cells. The *scale bar* indicates 2 µm. **c** Desmosomes were found between the cells (*arrowheads*). *Arrows* show cytokeratin bundles. The *scale bar* indicates 200 nm

TE-10 cells had accumulated between 3 and 5 layers by 24 h after plating in the holes of silicone blocks, as described in the Materials and Methods section. Desmosomes were observed between cells by transmission electron microscopy (Fig. 1b, c; Additional file 1). Although obvious basal laminae could not be observed and apical-basal polarity was morphologically vague between the cells of the upper and lower layers, we observed desmosomes, cytokeratin bundles, and microvilli in the uppermost layer of cells as signs of its differentiation into a stratified squamous epithelium.

In Hoechst 33342-stained specimens almost all of the visualized areas were characterized by overlapping nuclei, with the exception of the margin of the cell sheet (Fig. 1a). Thus, we concluded that TE-10 cells were able to form a stratified epithelial sheet under the growth conditions used.

### Modified scratch and scrape assays

The scratch assay is a simple and widely used method for inducing collective cell migration [32]; however, the margins of cell sheets frequently detach when scratch assays are performed with stratified epithelial sheets. Another common method involves using silicone stencils; this method results in milder damage to the cell sheet compared to the scratch assay, and some previous reports have demonstrated the effectiveness of silicone stencils on extracellular matrices. However, because we observed that the marginal cells of the stratified cell sheet were easily detached from the glass slides with silicone stencil use, we devised an improved method observe collective cell migration.

**Fig. 2:**
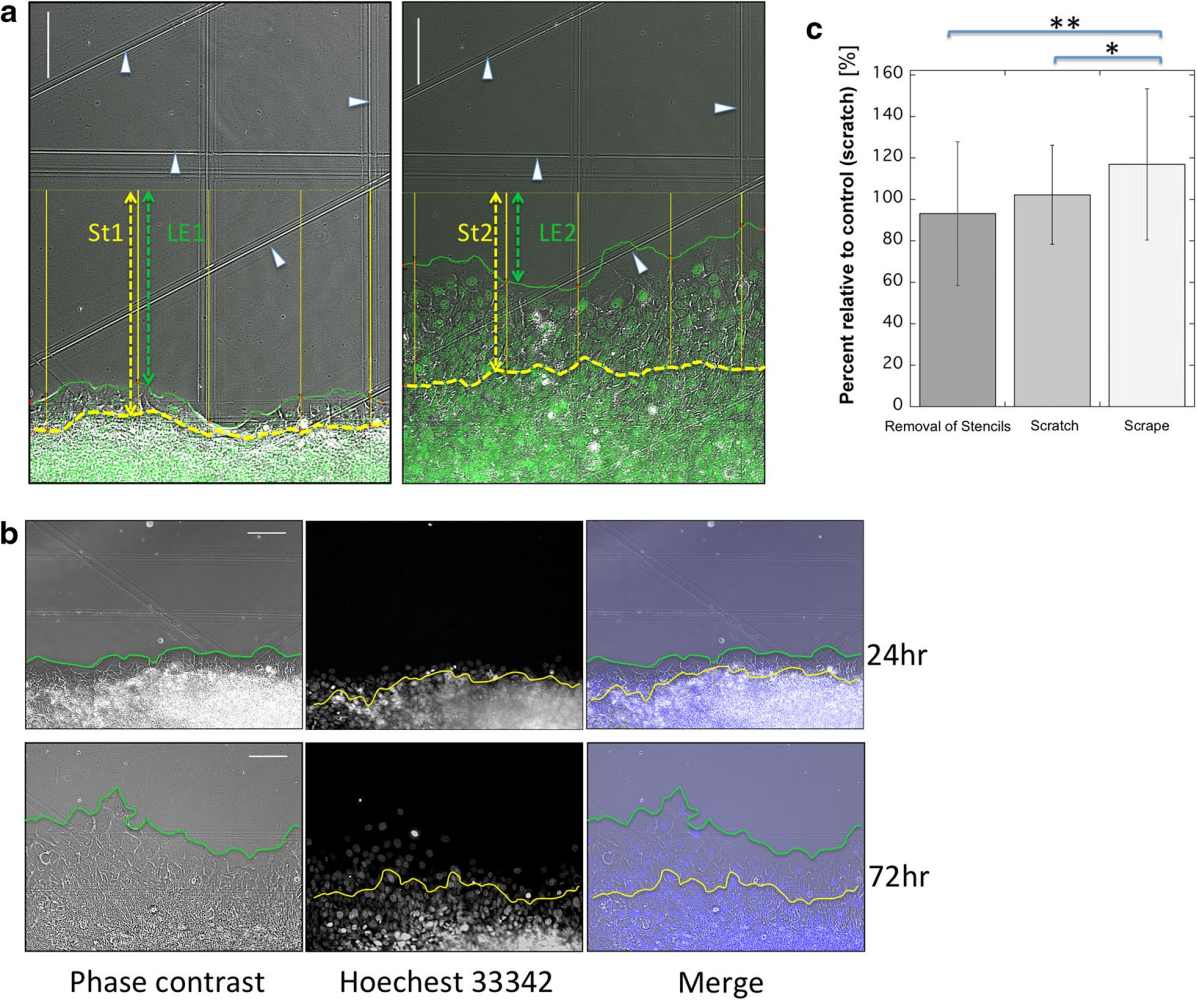
Migrating epithelia at 24 and 72 h after scraping the same area. **a** Photomicrographs demonstrating the method used to measure the distance of migration of epithelial cells. Four scratches on the glass were used as coordinates (*arrowheads*). The *photograph* on the *left* was taken 24 h after scraping, and the *photograph* on the *right* was taken at 72 h. The epithelial front is indicated by the *green line*, and the margin of the stratified region is indicated by the *blue line*. Both the epithelial front and the margin of the stratified region migrated upward. The distances of migration for both the migrating front and the stratified region were measured along randomly selected *lines* (5 *yellow lines*) perpendicular to the initial *start line* (generated by scraping), and the average moving distance was calculated. The change in the leading edge (LE) was calculated as ΔLE = LE1 − LE2, and the change in the stratified region was calculated as ΔSt = St1 − St2. **b** The migrating epithelia at 24 and 72 h after scraping. All the *photographs* are of the same area. The epithelia migrated upward during this 48-h period. *Green line* wounded edge of the cell sheet. *Yellow line* front margin of the stratified region. *Left* phase-contrast micrographs. *Center* Hoechst-stained specimens. *Right* merged images. **c** Comparison of the average migrated distances between the three methods (removal of stencils, scratch, and scrape). The experiments were performed in triplicate, and each experiment included at least three samples studied with each method. The values obtained by the scratch method were used for the control, and the migration distances observed following stencil removal and the scrape method are shown as ratios relative to the control. *Error bars* indicate SEMs. *p < 0.001; **p < 0.017

We found that a sharp wound edge was easier to create using our novel scrape method (described in the “Methods” section) than when using the silicone stencil or scratch methods. Using our novel scrape method, we also found that collective cell migration proceeded faster than had been reported for either of the other 2 methods (Fig. 2c). These results suggest that the scrape method causes less damage to cells and is, therefore, a more suitable approach for inducing migration compared to the other methods investigated.

Our observations began at 24 h after wounding because the initial wounds could be unstable. Our results demonstrated that the wound edge changed structurally to form a single layer of cells (monolayer) as it advanced forward, while cells in the rear (5 or 6 rows behind the leading edge) remained stratified during forward migration, as shown by Hoechst 33342 staining (Fig. 2b; Additional files 2, 3). Thus, both the cells of the wounded edge and the stratified cells behind the wounded edge moved forward as a group.

In addition, time-lapse analysis indicated that the cells found along the edge of the cell sheet were maintained in a well-adhered state and did not detach from each other during the 24–72 h period after wounding (as seen in the movies in Additional files 4, 5, 6, 7, 8 and 9). Notably, mitosis was observed across almost of the entire wounded area, including at the leading edge of the wound. These observations were in contrast to a previous report, the results of which suggested that cell division rarely occurred in the leading row of cells [5].

### Relationship between velocity and cell number

No relationship was observed between the velocity of the wound edge and the number of cells plated. The volume of the cell suspension plated into silicone stencils varied from 100 to 200 μL (3.0 × 10^6^ cells/m), but the velocity of migration for the wounded edge did not change, indicating that velocity was independent of cell number (Additional file 10). A 60-mm petri dish has an average TE-10 cell density of 0.05 × 10^4^ cells/mm^2^ at confluence; therefore, the cell density in the hole of the silicone block was 2.7-times higher than that in a confluent petri dish when 100 µL of the cell suspension was plated (Table 1), and the cells were highly overconfluent. This finding was also confirmed by Hoechst nuclear staining and electron micrographs, as described above. These results indicated that the cells moved actively in this stratified epithelial model rather than migrating through a passive mechanism such as gravity.

**Table 1:** Relationships between volumes plated inside the stencils, cell numbers, and cell concentrations

Volume (μL)	Cell number (×10^5^)	Relative to confluent cultures
100	3.0	2.72
150	4.5	4.09
200	6.0	5.45

The concentration of cells when 100 µL of cells were plated was approximately 3 times higher than that of confluent cultures. The relative concentration of cells when 200 µL of cells were plated was approximately 5-fold higher than that of confluent cultures

### Pharmacological inhibitors of ROCK influenced the collective migration and morphology of TE-10 cells

**Fig. 3:**
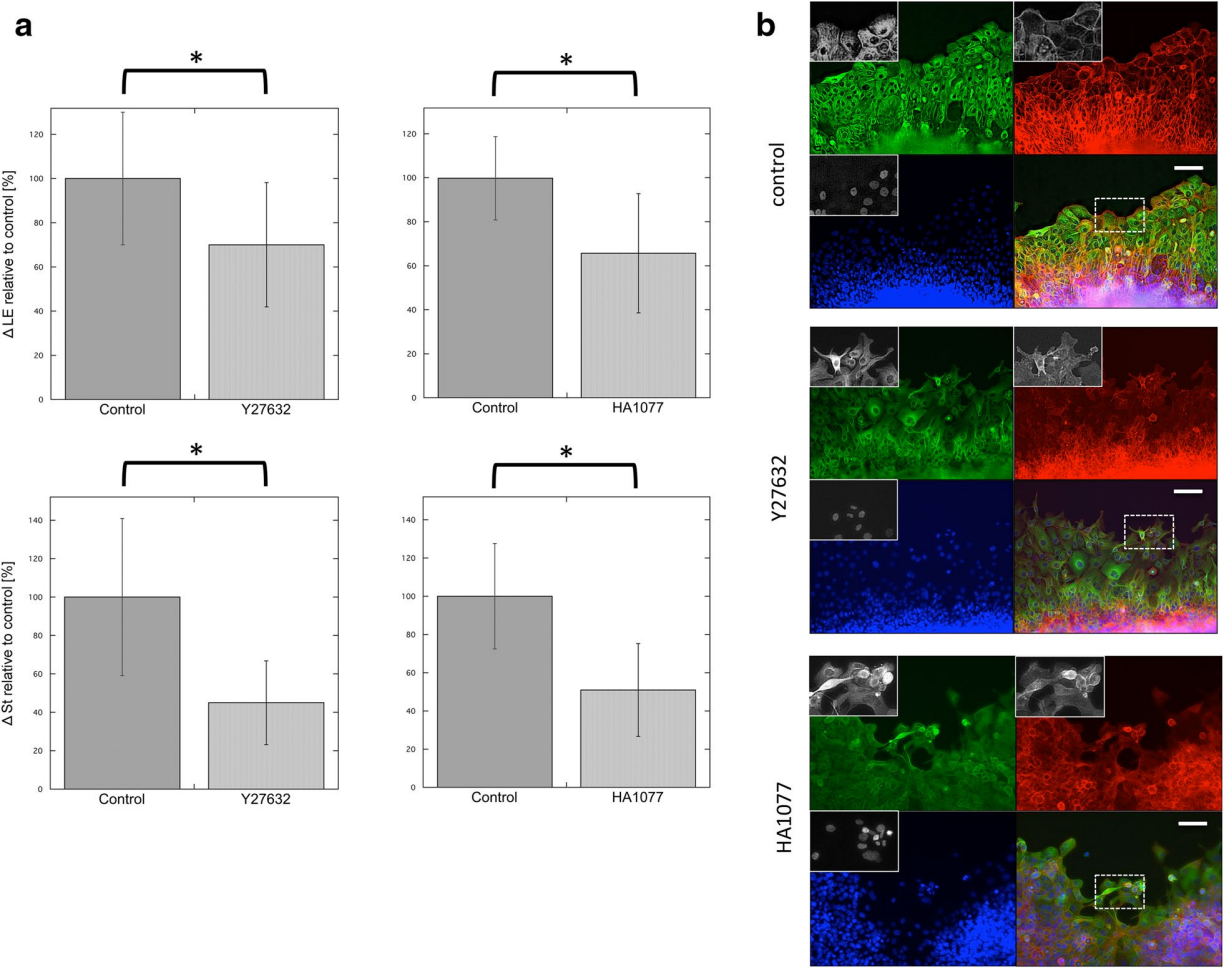
The effect of ROCK inhibitors on the collective cell migration of stratified TE-10 cells. **a** The distances of migration of cells found along the wound edges for each experimental condition (ΔLE) are shown in the *top panels* (data shown represent the mean ± SEM). Two inhibitors of ROCKs, Y27632 and HA1077, caused a significant reduction in migration (*p < 0.001). The *lower graphs* show the distances of migration for cells in the stratified region (ΔSt). Both inhibitors had a statistically significant influence on ΔSt (*p < 0.001). The data show the ratio of the migration distance relative to the control. All experiments were performed at least three times. **b** Images of the wounded edges of each cell sheet after various experimental treatments. *Top* control study. The cells in the *front row* were regularly arranged and well spread. *Middle* Cells treated with 5 µM Y27632. Many of the cells in the *front row* showed elongated shapes, with some protrusion of spikes. The arrangement of these cells appeared to be irregular, and the cell sheets were fragmented. *Bottom* Images of the cells found along the wounded edge after treatment with HA1077. Some of the cells in the leading row were small and round, while others were elongated and similar to the cells treated with Y27632. Empty space was observed between cells, a phenomenon that was not seen in control experiments. *Green* α-tubulin, *red* β-actin, *blue* nuclei; *scale bar* 100 µm. Each *inset* shows the magnified image of the area surrounded by the interrupted *white line* on the merged images

Both Y27632 and HA1077, well-known ROCK inhibitors [33, 34], reduced the distance that the wound edge moved at 24–72 h post-scraping (Fig. 3a; Additional file 11). In the stratified region of the cell sheet, migration was also reduced compared to that of control-treated cells (Fig. 3a). The results indicated that ROCKs affected the collective cell migration of TE-10 cells.

The morphology of the wound edge was also altered by treatment with ROCK inhibitors. Cell sheets along the leading edge were fragmented into many portions. Lamellipodia often became obscure, and the cell shapes were occasionally irregular at 72 h post-scraping (Fig. 3b). However, because of cell crowding and overconfluence, we were unable to evaluate the morphological changes occurring in cells at the rear.

Furthermore, the leading edge of TE-10 cells migrated in a disordered fashion when treated with Y27632 or HA1077, whereas well-regulated movement was observed in control TE-10 cells as shown in the movies in Additional files 12, 13, 14 and 15. These differences might be due to the disturbance of the cells along the wound edge upon treatment with ROCK inhibitors.

### Effects of knocking down ROCK expression

**Fig. 4:**
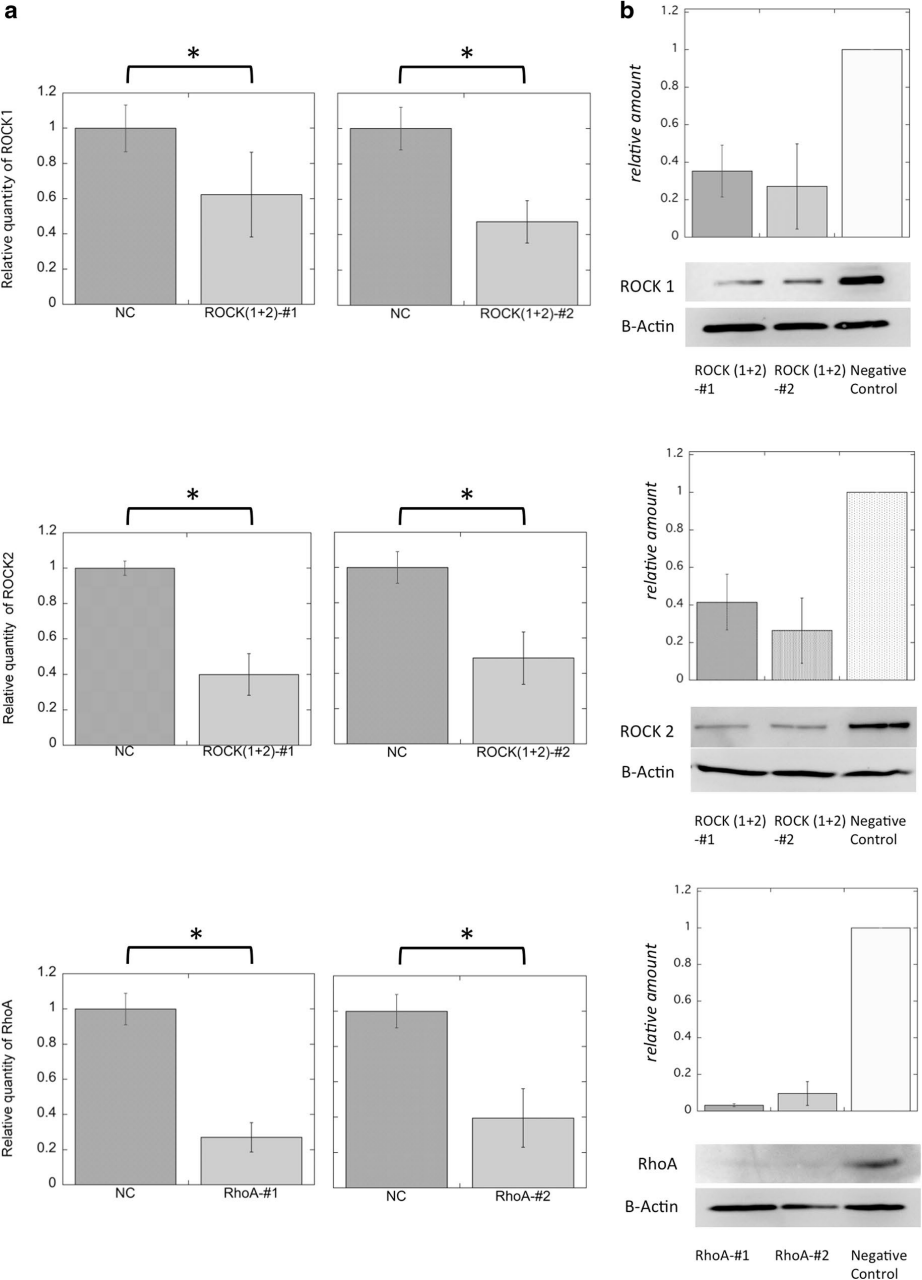
Efficiency of siRNAs targeting ROCKs and RhoA. **a** The effectiveness of each siRNA knockdown was evaluated by quantitative polymerase chain reaction analysis. The relative quantities of *ROCK1*, *ROCK2*, and *RhoA* mRNAs were compared between each experiment. *Top* *ROCK1* expression was reduced to about 60 and 40 % that of the control using ROCKs-#1 or ROCKs#2 siRNAs. *Middle* Both combinations of *ROCK* siRNAs reduced *ROCK2* mRNA levels to 40–50 % that of the control. *Bottom* Each *RhoA* siRNA reduced *RhoA* mRNA levels by about 40 % that of the negative control. Data represent the mean ± SEM of 3 independent experiments. *NC* negative control. Each *graph* indicates significant difference (*p < 0.01, n = 3). **b** Immunoblots showing protein expression of ROCK1, ROCK2, RhoA, and β-actin following the incubation of TE-10 cells with siRNA for each target. The expression of each protein was well suppressed, even after 24 h after RNAi treatment. Bars indicate the mean ± SEM of experiments performed in duplicate (RhoA) or triplicate (ROCKs)

**Fig. 5:**
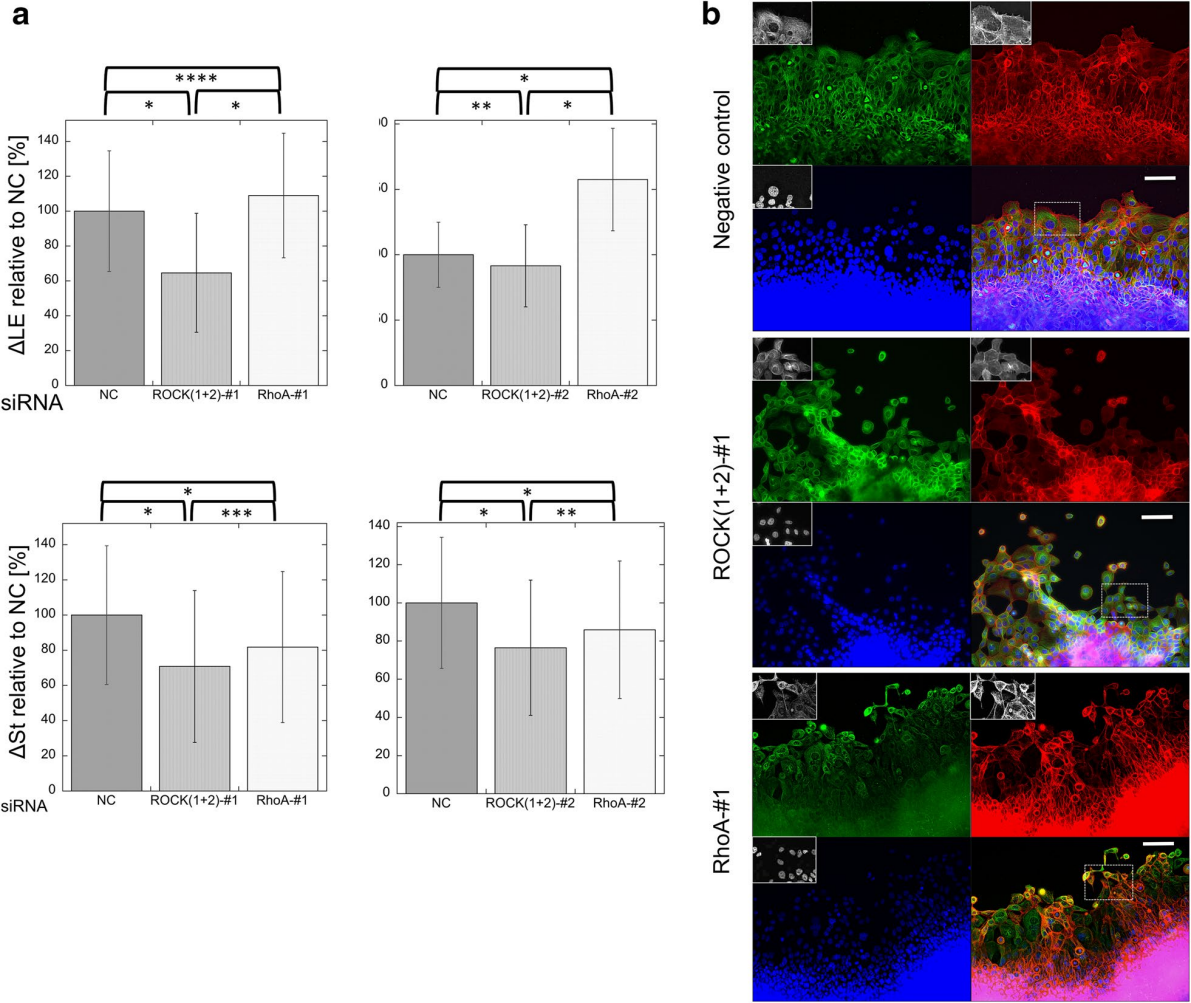
Migration of TE-10 cell sheets with siRNA knockdown of target mRNAs 72 h after scraping. **a** Cell migration was quantified over a 48-h period. For each experiment, negative control values were used as the reference. The *upper graphs* show the migration velocity of the leading edge (Δ LE), while the *lower graphs* show the velocity of the leading *line* of the stratified region. Data shown represent the mean ± SEM of at least six independent experiments in which at least three series were stained with Hoechst 33342 and at least 3 series were stained with H2B-GFP. *p < 0.001; **p = 0.002; ***p = 0.0028; ****p = 0.0044. ANOVA was performed using the original data. *NC* negative control. **b** Images of the cell sheets 72 h after scraping under siRNA treatment. *First row* negative control scrambled siRNA. The shape of the cells in the front row was very similar to that of untreated cells. There was no obvious irregularity in the arrangement of the cells. *Second row* knockdown of ROCKs by siRNA (ROCK1-#1 and ROCK2-#1). The arrangement of the cells in the leading row was obviously disordered. Many cells in the leading row were small with hypoplastic stress fibers. Several empty spaces between the cells were visible. *Third row* knockdown of RhoA by siRNA (RhoA-#1). The cells in the simple layer region were smaller than those in the negative control. Many of the cells were small and round, with inconspicuous stress fibers. *Green* α-tubulin, *red* β-actin, *blue* nuclei; *scale bar* 100 µm. Each *inset* shows the magnified image of the area surrounded by the interrupted white line on the merged image

Small interfering RNAs (siRNAs) targeting either ROCK1 or ROCK2 reduced the expression of the respective ROCK isoform mRNAs by approximately 40–60 %, compared to negative control cells transfected with a non-silencing siRNA (Fig. 4a). A combination of both siRNAs reduced the expression of ROCK1 and ROCK2 proteins by 70–90 % (Fig. 4b; Additional files 16, 17). Furthermore, knockdown of both ROCK1 and ROCK2 led to an estimated 20 % reduction in the migration distance of the wound edge (Fig. 5a). In cells transfected with ROCK siRNAs, the migration distance of the stratified region toward the rear of the wounded edge also significantly decreased by 20–30 % relative to negative control cells (Fig. 5a). In ROCK-knockdown cells, some cells along the leading edge assumed irregular shapes, and the lamellipodia became obscure, which was similar to the results observed with cells treated with ROCK inhibitors (Fig. 5b; Additional file 18, and the movies in Additional files 19 and 20).

### Effects of RhoA knockdown using siRNA

RhoA silencing with siRNA was performed as an alternative method to inhibit the function of ROCKs. Both RhoA siRNAs tested reduced RhoA RNA levels by about 40 % compared to negative control cells (Fig. 4a), and protein levels were reduced to 3–15 % (Fig. 4b; Additional file 21). In RhoA-knockdown TE-10 cells, the leading row of cells migrated approximately 10–40 % farther than did the negative control cells, a result that conflicted with that observed for ROCK inhibition (Fig. 5a). However, the stratified region moved only 80 % as far as the negative control. Furthermore, many cells along the leading row were irregularly shaped, the lamellipodia became obscure, and cells frequently separated, demonstrating reduced intercellular adhesion (Fig. 5b; Additional file 18, and the movies in Additional files 22 and 23).

### Relationship between the distance of cell migration and mitosis

**Fig. 6:**
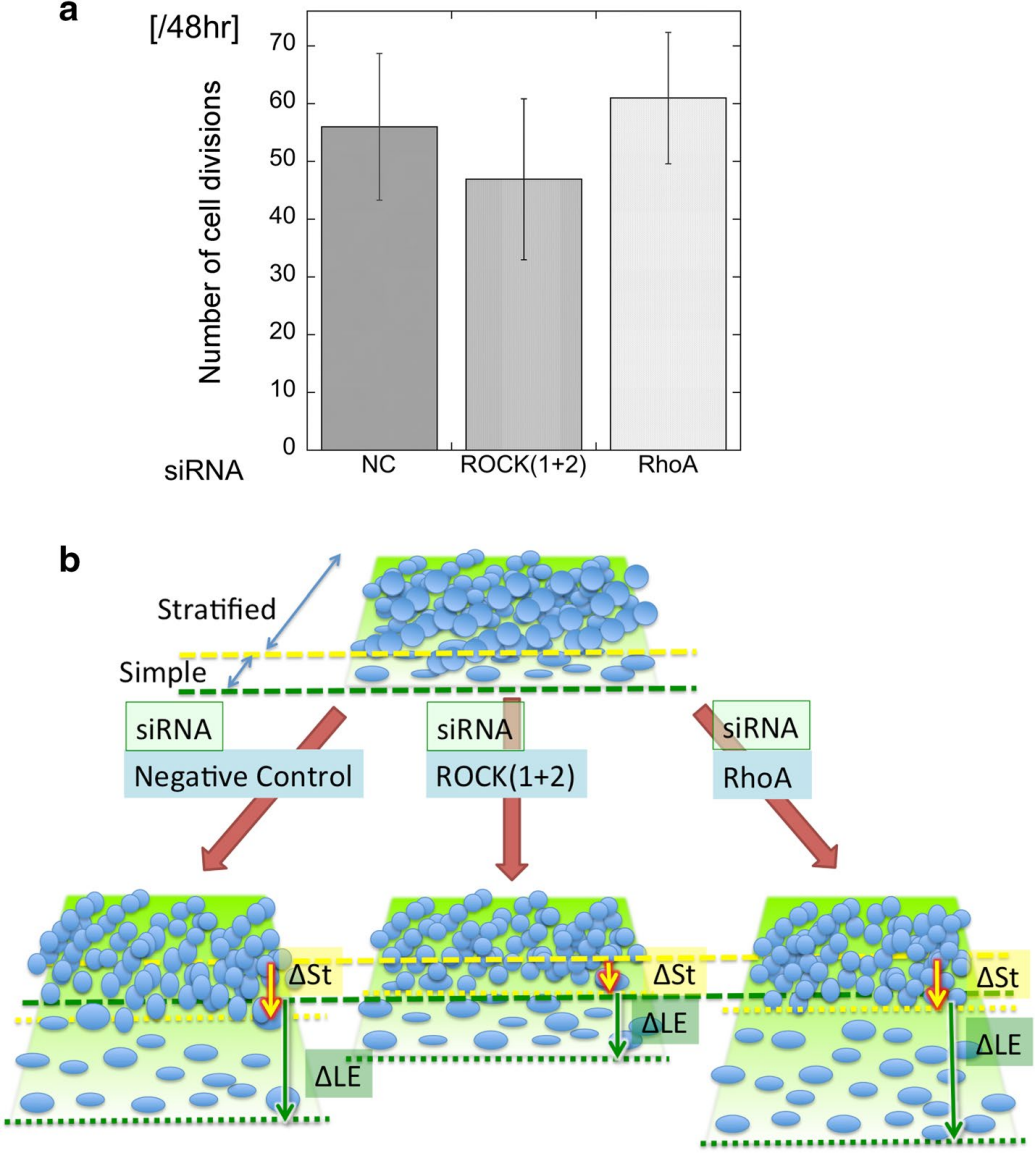
**a** Total numbers of mitotic cells, as determined by time-lapse video analysis. Data shown represent the mean ± SEM of at least three different visual fields. No significant differences were observed (ANOVA). **b** Schemes presenting the differences between ΔLE and ΔSt for each siRNA knockdown condition. The velocity of the leading wounded edge (ΔLE) of cells with siRNA-mediated knockdown of RhoA was faster than those of control cells and of cells with siRNA-mediated knockdown of ROCKs. The velocity of the stratified region (ΔSt) of ROCKs-knockdown cells was the slowest among all samples

Next, we examined the contribution of cell proliferation to the collective migration of TE-10 cells. The number of mitotic cells shown in movies of time-lapse analyses was compared between each experimental condition (siRNA-control, siRNA-ROCKs, and siRNA-RhoA). The results revealed no significant differences between these groups (Fig. 6a). Thus, differences in migration speeds were independent of cell proliferation rates, under the various experimental conditions tested here.

## Discussion

Several reports have demonstrated the formation and migration of stratified epithelial cell sheets using techniques such as culturing cells on an air–liquid interface [12, 35, 36], on specialized extracellular matrices [10, 13], and by using commercially available kits and protocols [37, 38]. These methods have limitations, such as long preparation times for the formation of stratified epithelia and the requirement for specialized devices to perform scratch assays. In the present study, we succeeded in creating stratified cell sheets with intercellular junctions from TE-10 cells by high-density culture in only 12 h (Additional file 1). In addition, collective cell migration was induced on glass slides using a scrape assay without any visible damage to the marginal cells. Whereas previous studies have demonstrated no significant difference in the migration rates between cells subjected to the classical scratch assay and those induced to migrate by the removal of silicone stencils [2], the results of this study revealed much more rapid collective cell migration using our novel scrape method. The increased migration rates of cells subjected to scrape assays might have been a consequence of the lower degree of damage to cells induced by this method.

In this study, at the start of the observation all of the leading edges were simple non-stratified monolayers. The formation of a monolayer at the leading edges might be a characteristic behavior of stratified epithelia in in vitro studies [39, 40]. Furthermore, the cell migration model used in this study revealed that active, rather than passive, cell migration occurred in the stratified epithelia. Our results also suggested that migration occurred via a “sliding”-type of movement where the position of the cells relative to the adjacent cells rarely changed during the movement, rather than a “leap-frog” type of movement in which the cells in the rear processed beyond the front row cells [41].

The effects of ROCKs on cell migration are complicated and have not yet been fully clarified, as they seem to be highly cell-type dependent [19, 42, 43]. In this study, the collective migration of TE-10 cells was significantly inhibited by each of 2 different ROCK inhibitors and by 2 combinations of siRNAs targeting ROCKs, indicating an important role for ROCKs in this process. This conclusion is consistent with predictions based on clinical specimens of esophageal cancer where the overexpression of RhoA mRNA or protein correlated with tumor differentiation, depth of invasion, and worsened prognosis [31, 44]. In addition, ROCK inhibition appeared to affect cell morphology, as protrusions were observed in many of the cells found along the leading edge row in experiments using both ROCK inhibitors and ROCK siRNAs. This observation is also consistent with previous studies [20, 45, 46].

Notably, siRNA-mediated knockdown of RhoA, an upstream regulator of ROCKs, caused a significant increase in the migration speed of the leading row of cells (Figs. 5a, 6b), which was inconsistent with the results of our experiments using ROCK inhibition. Concomitantly, the migration speed of the stratified area in the RhoA knockdown cells decreased significantly. These conflicting results might be explained by one of several possibilities. One possibility is that in our experimental system, ROCKs did not serve as downstream effectors of RhoA, but were members of another signaling system. However, this seems unlikely given that the RhoA-ROCKs signaling pathway has been widely demonstrated in various experimental systems. Another possibility is that RhoA silencing might have inhibited the downstream activation not only of ROCKs but also of additional downstream RhoA effectors that normally block the functions of ROCKs. For example, mDia1 is a downstream effector of RhoA that participates in a different signaling system than the RhoA-ROCKs pathway and inhibits the activity of ROCKs under certain conditions [47]. We consider the latter hypothesis to be the most plausible (Additional files 17, 18, 20).

In addition, the effectiveness of suppression of protein expression by RNA interference (RNAi) should be considered (Additional files 21, 22, 23). It is possible that ROCK proteins might show extremely different effects on cell movement depending on their concentrations. Recently, Schofield et al. reported a novel ROCKs signaling pathway that regulates cell migration, using a series of sophisticated experiments to demonstrate ROCKS activity [22]. In this study, we showed that ROCK protein levels were partially blocked by RNAi knockdown of ROCK expression; however, we could not determine the extent of the functional inhibition of ROCKs in the RhoA RNAi experiments. We hope to clarify the originally confusing results obtained in the RhoA knockdown TE-10 cells through additional ongoing experiments, including determining the status of ROCKs activity.

The compositions of monolayer and stratified portions differ, in that the former is composed only of basal cells whereas the latter is composed of both basal and suprabasal cells. The adhesion mechanisms, which mediate migratory processes, differ between the basal layer and suprabasal cells: whereas basal cells form contacts with both extracellular matrices and adjacent cells, suprabasal cells form only the latter contacts [48]. Furthermore, although both cell–cell and cell–matrix junctions play important roles in cell migration, their composite molecules differ. Thus, they might react differently to the suppression of members of the Rho signaling pathway and its disruption between the cells in the simple layer and those in the stratified portion. In addition, further confounding this issue, we were not able to distinguish between the basal and suprabasal cells in the stratified portion and trace the movement of basal cells in this area.

While some researchers have postulated that both the speed and doubling times of individual cells exert little influence on collective cell migration [49], a few studies have suggested that mitosis can affect collective cell migration [50, 51]. In our study, the doubling time of the cells found along the wound edge might have differed from that of the cells located within the central region of cell sheets. Furthermore, it was difficult to select comparable areas between experiments because the migration velocities were altered under the varying experimental conditions. However, our data suggested that the rate of mitosis did not significantly affect the rate of collective cell migration, as no differences in the frequencies of mitotic cells at the wound edges were observed between the different treatments. Further studies are required in order to gain a better understanding of the mechanisms involved in these processes.

## Conclusions

We demonstrated that stratified epithelial cell sheets of TE-10 cells formed quickly using a relatively simple novel method. ROCKs appeared to play an important role in mediating the migration of this cell sheet. Furthermore, our data suggested that differences exist between the mechanisms of collective cell migration in the single-layer region of the wound edge and in the stratified layer of the following region.

## Methods

### Inhibitors

The following ROCK inhibitors were used in this study: Y-27632 (20 μM; Sigma-Aldrich, St. Louis, MO, USA) and hydroxyfasudil (HA1077, Fas-OH; 5 μM; Wako, Osaka, Japan) [52].

### Nuclear staining

Hoechst 33342 (1 mg/mL) was purchased from Dojindo Laboratories (Kumamoto, Japan) and was used at a 1:1000 dilution for nuclear staining. Propidium iodide (PI; 1 mg/mL) was also purchased from Dojindo Laboratories and was used at a 1:100 dilution for nuclear staining of nonviable cells.

### Primary antibodies

Monoclonal mouse anti-human α-tubulin (T6199; Sigma-Aldrich, St. Louis, MO, USA) was used at a 1:500 dilution for microtubule staining [53, 54].

### Secondary antibodies

Alexa Fluor 488- and Alexa Fluor 546-conjugated goat anti-mouse IgG antibodies (A11017 and A11030; Invitrogen, Carlsbad, CA, USA) were used as secondary antibodies at a 1:100 dilution.

### Stencils

Stencils were molded in polydimethylsiloxane (PDMS) elastomers (Sylgard 184; Dow Corning, Midland, MI, USA). Monomers of the elastomers were mixed with curing agents and poured into a plastic dish to prepare an 8 mm thick sheet. The cross-linked PDMS sheet was then slowly removed and was formed into 1.5 cm^2^ blocks. An 8 mm diameter hole was punched in the center of the block, and the block was then washed in detergent (Nalgene L900) for several hours, rinsed, air dried, and sterilized by autoclaving directly before use.

### Cell culture

TE-10 cells, derived from a well-differentiated human esophageal squamous cell carcinoma, were obtained from the Riken Bioresource Center (Tsukuba, Japan) [55–57]. TE-10 cells were maintained in Dulbecco’s modified Eagle medium **(**DMEM) containing 10 % fetal bovine serum (FBS), 0.03 % glutamate, and 0.6 μg/mL kanamycin and were grown at 37 °C with 5 % CO_2_ in a humidified tissue culture incubator. For certain experiments, TE-10 cells were transfected with an expression vector encoding H2B-GFP (Plasmid 11680; Addgene, Cambridge, MA, USA), according to the manufacturer’s protocol. Following limiting dilution, individual colonies were transferred to new tissue culture plates using cloning disks. Clones expressing high levels of GFP-H2B were selected for experimental use.

### Stratifying technique: high-concentration cultures

PDMS stencils were attached to glass plates (21.5 × 26 mm) made from glass slides. Fine lines were drawn on the surface of the plates as guidelines for observation, and the plates were placed in 35 mm petri dishes.

TE-10 cells that were more than 80 % confluent were trypsinized, collected, and centrifuged. The supernatant was discarded, and the cells were resuspended in medium at a density of 3.0 × 10^6^ cells/mL. Cells were seeded into the hole in the center of each PDMS stencil using a micropipette. The volume of cells seeded was normally 150 μL unless otherwise stated. Stencils were incubated for 24 h at 37 °C in a 5 % CO_2_ incubator.

### Scratch and scrape wounding assays

Scratch assays were performed using standard procedures [32]. Briefly, stencils were carefully removed after incubation. The stratified cell sheet was scratched in a straight line with a 20 μL plastic pipette tip to create a cell-free gap. Thereafter, the medium was replaced with phosphate buffered saline (PBS: 1X) containing Hoechst 33342 for nuclear staining for 5 min. After staining, cells were rinsed with PBS 3 times, and 2 mL culture medium was added.

Scrape assays were performed using flame-sterilized slide glass to scrape off approximately half of the cell sheet, and then the stencil was removed. The cell sheet remaining on the glass was rinsed with PBS three times and placed in an incubator after the addition of 2 mL culture medium.

Cell movement was visualized using a fluorescent microscope (Biorevo BZ9000; Keyence, Osaka, Japan). Images were taken in phase-contrast mode, and complementary images of nuclei were obtained. At least 3 positions per dish were recorded at 24, 48, and 72 h after the cell sheets were scratched or scraped. The average distances of migration for both the migrating front and the stratified region were measured along randomly selected lines perpendicular to the initial start line generated by scraping the surface of the glass (Fig. 2a). Time-lapse images were taken using automated time-lapse microscopy, performed with a Leica FW4000 microscope (Leica Microsystems, Wetzlar, Germany) at 37 °C and 5 % CO_2_. Both phase-contrast images and fluorescence images were taken every 15 or 30 min for 48 h beginning at 24 h post-wounding.

Image analysis was performed using software provided with each microscope or ImageJ software (1.45 s; National Institute of Health, Bethesda, MD, USA). Statistical analyses were performed using KaleidaGraph (version 4.1; Synergy Software, Tokyo, Japan). Cell viability was confirmed by PI staining after certain time-lapse image experiments (data not shown).

### Immunostaining and F-actin staining

TE-10 cells were fixed on glass plates with periodate lysine paraformaldehyde and permeabilized in 0.1 % Triton X-100 (Sigma-Aldrich, Tokyo, Japan). After incubation with blocking medium (5 % bovine serum albumin in PBS), samples were incubated with primary antibodies for 2 h at room temperature, according to the manufacturer’s instructions. Cells were then rinsed 3 times with PBS and incubated with Alexa Fluor 488- or 546-conjugated secondary antibodies, according to the manufacturer’s instructions. To detect actin, cells were stained with Alexa Fluor 546-conjugated phalloidin (A22283; Invitrogen). Fluorescent images were obtained under a fluorescence microscope or a laser-scanning confocal microscope (LSM510; Carl Zeiss, Tokyo, Japan).

### siRNA knockdown of ROCK and RhoA

Two 21-mer duplex chimera-type siRNAs targeting ROCK 1, ROCK2, or RhoA were selected and obtained from the website library of RNAi Co. Ltd. (Tokyo, Japan; https://sidirect.jp/esd/modules/modsiperfect/). Cells were transfected with siRNA duplexes (100 nM final concentration, Additional file 24) using Lipofectamine RNAiMAX (Invitrogen) for 72 h at 37 °C in a CO_2_ incubator, following the manufacturer’s instructions. A non-silencing control sequence (RNAi Co. Ltd.) was used as a control (100 nM final concentration). Sequences of the siRNA constructs are shown in Table 2.

**Table 2:** Sequences of chimeric siRNAs used in this study

	5′-	3′-
Rock1-#1	CGA CUG GGG ACA Gtt ttg aga	tca aaa CUG UCC CCA GUC GAC
Rock2-#1	GUU AGU CGG UUG Gtg aaa aag	ttt tca CCA ACC GAC UAA CCC
RhoA-#1	GUU AGU UAC CUU Ata gtt act	taa cta UAA GGU AAC UAA CAU
Rock1-#2	CCA AAG CUC GUU Uaa ctg aca	tca gtt AAA CGA GCU UUG GUU
Rock2-#2	GUU GGA ACU AAU Ata tcc ttg	agg ata UAU UAG UUC CAA CAC
RhoA-#2	GAU UAU UAA CGA Ugt cca acc	ttg gac AUC GUU AAU AAU CAU
Negative control	GUA CCG CAC GUC Att cgt atc	tac gaa UGA CGU GCG GUA CGU

Capital letters indicate RNA sequences, while small letters indicate DNA sequences

### Quantitative real-time reverse transcription PCR (qRT-PCR)

After the indicated treatments, total RNA was isolated from cells using the TRI Reagent Kit (Sigma) and quantified using a UV spectrophotometer. RNA (1.5 µg/sample) was converted to cDNA by reverse transcription using the iScript cDNA Synthesis Kit (Bio-Rad Laboratories, Berkeley, CA, USA). The primers used for qRT-PCR were as follows: human glyceraldehyde-3-phosphate dehydrogenase (*GAPDH*), forward 5′-CTC TGA CTT CAA CAG CGA CAC-3′ and reverse 5′-CCT TGG AGG CCA TGT G-3′; human *ROCK1*, forward 5′-CGA AGA TGC CAT GTT AAG TGC-3′ and reverse 5′-ATC TTG TAG AAA GCG TTC GAG-3′; human *ROCK2*, forward 5′-TTA AGC CTC CTC CTG CTT TG-3′ and reverse 5′-CAC CAA CCG ACT AAC CCA CT-3′; and human *RhoA*, forward 5′-TAT CGA GGT GGA TGG AAA GC-3′ and reverse 5′-TTC TGG GGT CCA CTT TTC TG-3′. All primer sets were obtained from Sigma-Aldrich.

cDNA was amplified in a CFX96 Touch Real-Time PCR Detection System (Bio-Rad), and PCR products were detected by labeling with SsoFast EvaGreen Supermix (Bio-Rad). Amplification was performed using the following thermocycling conditions: initial denaturation for 30 s at 95 °C; 50 cycles each of denaturation for 3 s at 95 °C and annealing for 5 s at 55.0 °C; and a final extension for 10 s at 95 °C. *GAPDH* was used as an internal control to normalize gene expression levels between samples.

Duplicate samples were analyzed for each condition, and experiments were repeated using 3 independent mRNA preparations. Differences between treatments with respect to relative expression were expressed in terms of mean ± standard error.

### Western blotting

TE-10 cells transfected with siRNA were trypsinized and collected at 24 h post-treatment. After the density of each cell suspension was adjusted, the cells were washed once with cold PBS, centrifuged at 13,040×*g* for 5 min at 4 °C, and lysed with RIPA buffer. Cell homogenates were incubated for 2 min at 100 °C in 2× loading buffer and subjected to SDS-PAGE and western blot analysis, using Full-Range Rainbow Molecular Weight Markers (GE Healthcare Japan, Tokyo, Japan) as standards. After the proteins were transferred to polyvinylidene fluoride membranes (Millipore, Billerica, MA, USA), membranes were blocked for 2 h in Tris-buffered saline (TBS: 150 mM NaCl, 50 mM Tris, pH 7.4) containing 5 % fat-free dried milk. The following primary antibodies were used: rabbit anti-human ROCK1 (1:250; HPA007567; Atlas Antibodies, Stockholm, Sweden), anti-human ROCK2 (1:250; HPA007459; Atlas Antibodies), and anti-human-RhoA (1:100; 26C4; Santa Cruz Biotechnology, Dallas, TX, USA). Then, the membranes were incubated with horseradish peroxidase-conjugated secondary antibodies (1:2000; MP Biomedicals, Santa Ana, CA, USA) and immunoreactivity was visualized with the LAS-3000 Mini System (Fuji Photo Film, Tokyo, Japan). Where indicated, immunoblots were stripped with Re-Blot Plus Mild Antibody Stripping Solution (Millipore), according to the manufacturer’s protocol. The immunoblots were then re-probed with β-actin primary antibodies (1:2000; Santa Cruz Biotechnology) and horseradish peroxidase-conjugated secondary antibodies, and immunoreactivity was visualized with the LAS-3000 mini system. Densitometry was performed using ImageJ software, with normalization to the amount of cellular β-actin present in each sample.

### Transmission electron microscopy

Transmission electron microscopy images were captured as described previously [58]. The cells were fixed with 1.25 % glutaraldehyde and 1 % paraformaldehyde in 0.1 M phosphate buffer (pH 7.4), post-fixed with 1 % osmium tetroxide in the buffer, dehydrated with a graded series of ethanol, substituted by propylene oxide, and embedded in Epon 812. Ultrathin sections were prepared from polymerized Epon blocks with a Reichert Ultracut N Ultramicrotome (Leica Microsystems), stained with 2 % uranyl acetate in 70 % ethanol and 0.4 % lead citrate, and photographed using an H-7500 transmission electron microscope (Hitachi, Tokyo, Japan) operated at 80 kV.

### Statistical analysis

Each experiment was performed at least 3 times unless otherwise noted. Statistical analyses were carried out using KaleidaGraph version 4.1 by ANOVA or *t* test. All data are presented as means (±SEM). *p*-values <0.05 were considered to be statistically significant.

## Additional files

Additional file 1. Cross section of stratified TE-10 cells 12 h after seeding at high density. (**a**) Cross section images were taken using a transmission electron microscope and were reconstructed (PowerPoint for Mac 2011 version 14.5.2; Microsoft, Redmond, WA, US). TE-10 cells were seeded in a silicone stencil and incubated for 12 h before fixation. The cells were stratified into 3–5 layers in this specimen. The left side shows the basal layer, while the right side shows the apical layer, on which only a few microvilli are seen. Scale bar: 2 µm. (**b**) Desmosomes (arrows) were found in the specimen 24 h after plating. Scale bar: 500 nm. Additional file 2. Reconstructed images of the wounded edge 72 h after scraping. Images were taken with a laser scanning confocal microscope (LSM510; Carl Zeiss, Tokyo, Japan). The cells 5–7 rows behind the wounded edge were observed to be stratified. Red: f-actin, green: microtubules, blue: nuclei. Additional file 3. Images of a randomly selected point along the TE-10 cell sheet. (**a**) Twenty-four hours after scraping, a simple layer was observed in the first 4–5 rows of the wounded edge, followed by a stratified layer. (**b**) Seventy-two hours after scraping, the simple layer was observed in the first 4–6 rows of the wounded edge, while the stratified region had also advanced forward over a 48-h period, similar to the wounded edge. As shown in the H2B-GFP nuclear stained images, the simple layer was observed along the wounded edge followed by the stratified layer, similar to the results shown in Fig. 2. Scale bar: 100 µm. Green line: the front line of the cell sheet. Yellow line: the front border of the stratified layer. Additional file 4. Movies showing the movement of control cells treated with vehicle only. Movies reconstructed with phase contrast images. Additional file 5. Movies showing the movement of control cells treated with vehicle only. Movies reconstructed with images of nuclear staining. Green fluorescence was observed after transfecting cells with an expression vector encoding H2B-GFP. Additional file 6. Movies showing the movement of control cells treated with vehicle only. Movies reconstructed with phase contrast images. Additional file 7. Movies showing the movement of control cells treated with vehicle only. Movies reconstructed with images of nuclear staining. Green fluorescence was observed after transfecting cells with an expression vector encoding H2B-GFP. Additional file 8. A movie showing the movement of negative-control cells in an siRNA experiment. Movies reconstructed with phase contrast images. Additional file 9. A movie showing the movement of negative-control cells in an siRNA experiment. Movies reconstructed with images of nuclear staining. Green fluorescence was observed after transfecting cells with an expression vector encoding H2B-GFP. Additional file 10. Relationship between cell numbers and the velocity of the wounded edge. The volume of the cell suspension plated into the hole in the center of each PDMS stencil was increased in 50-µL increments from 100 to 200 µL. Distances migrated during the 48-h period were measured as described in the Materials and Methods section. Experiments were performed in triplicate, and at least 3 samples were studied for each volume plated. Ratios relative to the data for the 150-µL volume are shown as means ± SD. No significant differences were observed. Additional file 11. Effects of a Rac1 inhibitor on the collective migration of TE-10 cells. A series of scrape assays was performed using NSC23766, a well-known inhibitor of Rac1, at a final concentration of 50 µM. Only vehicle was added to the control samples. Data represent the mean ± SEM of the migration distance of the leading edge (ΔLE), using the original data. No significant differences were observed, as determined by a *t*-test (*p* = 0.227). Three different samples were prepared for each condition. Additional file 12. A movie showing the movement of cells treated with the ROCKs inhibitor Y27632. Movies reconstructed with phase contrast images. Additional file 13. A movie showing the movement of cells treated with the ROCKs inhibitor Y27632. Movies reconstructed with images of nuclear staining. Green fluorescence was observed after transfecting cells with an expression vector encoding H2B-GFP. Additional file 14. A movie showing the movement of cells treated with the ROCKs inhibitor HA1077. Movies reconstructed with phase contrast images. Additional file 15. A movie showing the movement of cells treated with the ROCKs inhibitor HA1077. Movies reconstructed with images of nuclear staining. Green fluorescence was observed after transfecting cells with an expression vector encoding H2B-GFP. Additional file 16. Original immunoblotting data for ROCK1 and β-actin. (**a**) The bands of approximately 160 kDa represent ROCK1, while other non-specific bands of approximately 45 kDa are those of β-actin. (**b**) The bands of approximately 45 kDa represent β-actin. (**c**, **d**) The dots on the sheet indicate the positions of each marker. The 150 and 102 kDa markers are obscure, while the yellow, 76 kDa band is relatively clear. The results of the second and the third series of experiments are shown. Additional file 17. Original immunoblotting data for ROCK2 and β-actin. (**a**) The clear bands approximately 160 kDa in size represent ROCK2, while the origin of the band at approximately 52 kDa is unknown. (**b**) The strong bands of approximately 45 kDa in size originated from β-actin. (**c**, **d**) The dots on the sheet show the positions of each marker. The 150 kDa and 102 kDa markers are obscure in the picture, similar to their appearance in Additional file 10. The results of the second and the third series of experiments are shown. Additional file 18. Images of TE-10 cell sheets with ROCK or RhoA siRNA knockdown 72 h after scraping. (**a**) TE-10 cells were transfected with siRNAs targeting ROCKs (ROCK1-#2 and ROCK2-#2). The irregular arrangement of the leading row of cells is shown, which was similar to that observed when cells were transfected with *ROCK1* siRNA-#1 and *ROCK2* siRNA #1. Stress fibers in the leading edge cells were also hypoplastic, although some cells were as large as the negative control cells. (**b**) TE-10 cells transfected with siRNA #2 targeting RhoA. Spaces between the cells were observed in the simple layer region. The cells in this region were small, with inconspicuous stress fibers, and there were few well-spread, fan-shaped cells. Green: α-tubulin, red: β-actin, blue: nuclei. Each inset shows the magnified image of the area surrounded by a white line on the merged image. Scale bar: 100 µm. Additional file 19. A movie showing the movement of cells transfected with siRNA-ROCKs#1. Movies reconstructed with phase contrast images. Additional file 20. A movie showing the movement of cells transfected with siRNA-ROCKs#1. Movies reconstructed with images of nuclear staining. Green fluorescence was observed after transfecting cells with an expression vector encoding H2B-GFP. Additional file 21. Original immunoblotting data for RhoA and β-actin. (**a**) The clear bands of approximately 21 kDa represent RhoA; obscure bands at approximately 21 kDa were observed following RhoA siRNA treatment. (**b**) The strong bands of approximately 45 kDa in size are β-actin. The white dots reflect the positions of the Rainbow Molecular Weight Markers (GE Healthcare Japan, Tokyo, Japan). (**c**, **d**) The dots on the sheet indicate the positions of each marker. The 150 kDa and 102 kDa markers appear obscure, similar to their appearance in Additional file 10. The results of the second and the third series of experiments are shown. Additional file 22. A movie showing the movement of cells transfected with siRNA-RhoA#2. Movies reconstructed with phase contrast images. Additional file 23. A movie showing the movement of cells transfected with siRNA-RhoA#2. Movies reconstructed with images of nuclear staining. Green fluorescence was observed after transfecting cells with an expression vector encoding H2B-GFP. Additional file 24. Viability of TE-10 cells treated with 200 nM siRNA. To investigate the toxicity of high dose siRNA and the viability of the TE-10 cells treated with 200 nM siRNA, ΔLE and ΔSt of 200 nM siRNA treatment of the negative controls were measured and compared with those of control cells treated with 100 nM siRNA. The data of ΔLE and ΔSt of each group were evaluated based on the mean of those of the 100 nM siRNA treated cells, whereas ΔSt/ΔLE were calculated using the original data of each category. The stratified layer of the cells treated with 200 nM siRNA showed slightly faster mobility than the cells treated with 100 nM (** p = 0.005), while there were no significant differences in ΔLE (* *p* = 0.375) and ΔSt/ΔLE (*** *p* = 0.136).

## Abbreviations

DMEM: Dulbecco’s modified Eagle medium
FBS: fetal bovine serum
GAPDH: glyceraldehyde-3-phosphate dehydrogenase
GFP: green fluorescent protein
GTP: guanosine triphosphate
H2B: histone 2B
PBS: phosphate buffered saline
PDMS: polydimethylsiloxane
PI: propidium iodide
qRT-PCR: quantitative real time reverse transcription polymerase chain reaction
RNAi: RNA interference
ROCK: Rho-associated coiled coil-containing protein kinases
siRNA: small interfering RNA
TBS: Tris-buffered saline
