# Supplementary figures and images for: Inhibition of Rho-associated kinases disturbs the collective cell migration of stratified TE-10 cells
Source: Biol Res. 2015 Sep 2;48(1):48. doi: 10.1186/s40659-015-0039-2 (PMC4556056; doi:10.1186/s40659-015-0039-2)

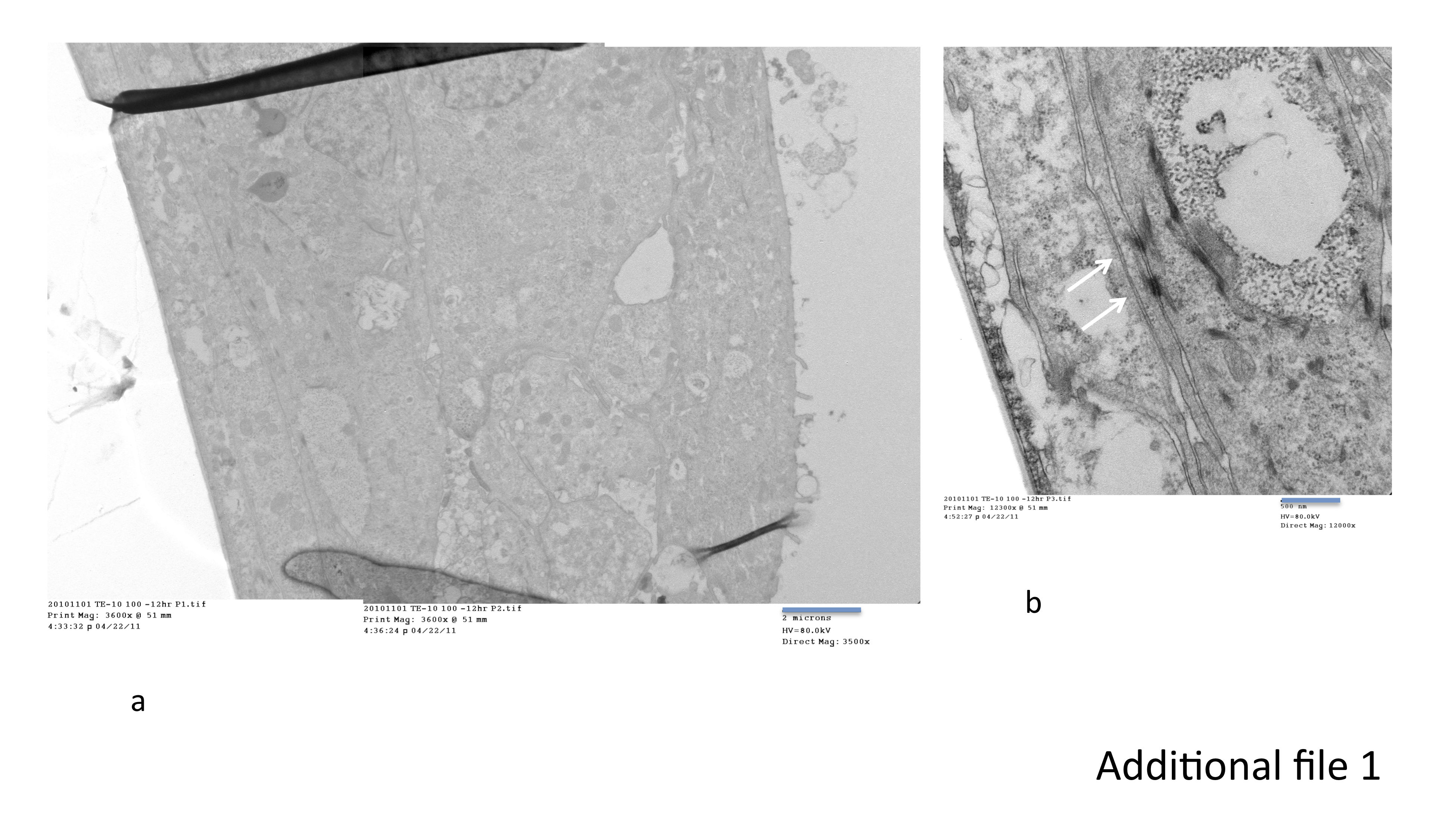

Supplement: Additional file 1. — Cross section of stratified TE-10 cells 12 h after seeding at high density. (a) Cross section images were taken using a transmission electron microscope and were reconstructed (PowerPoint for Mac 2011 version 14.5.2; Microsoft, Redmond, WA, US). TE-10 cells were seeded in a silicone stencil and incubated for 12 h before fixation. The cells were stratified into 3–5 layers in this specimen. The left side shows the basal layer, while the right side shows the apical layer, on which only a few microvilli are seen. Scale bar: 2 µm. (b) Desmosomes (arrows) were found in the specimen 24 h after plating. Scale bar: 500 nm. [file 40659_2015_39_MOESM1_ESM.tiff]

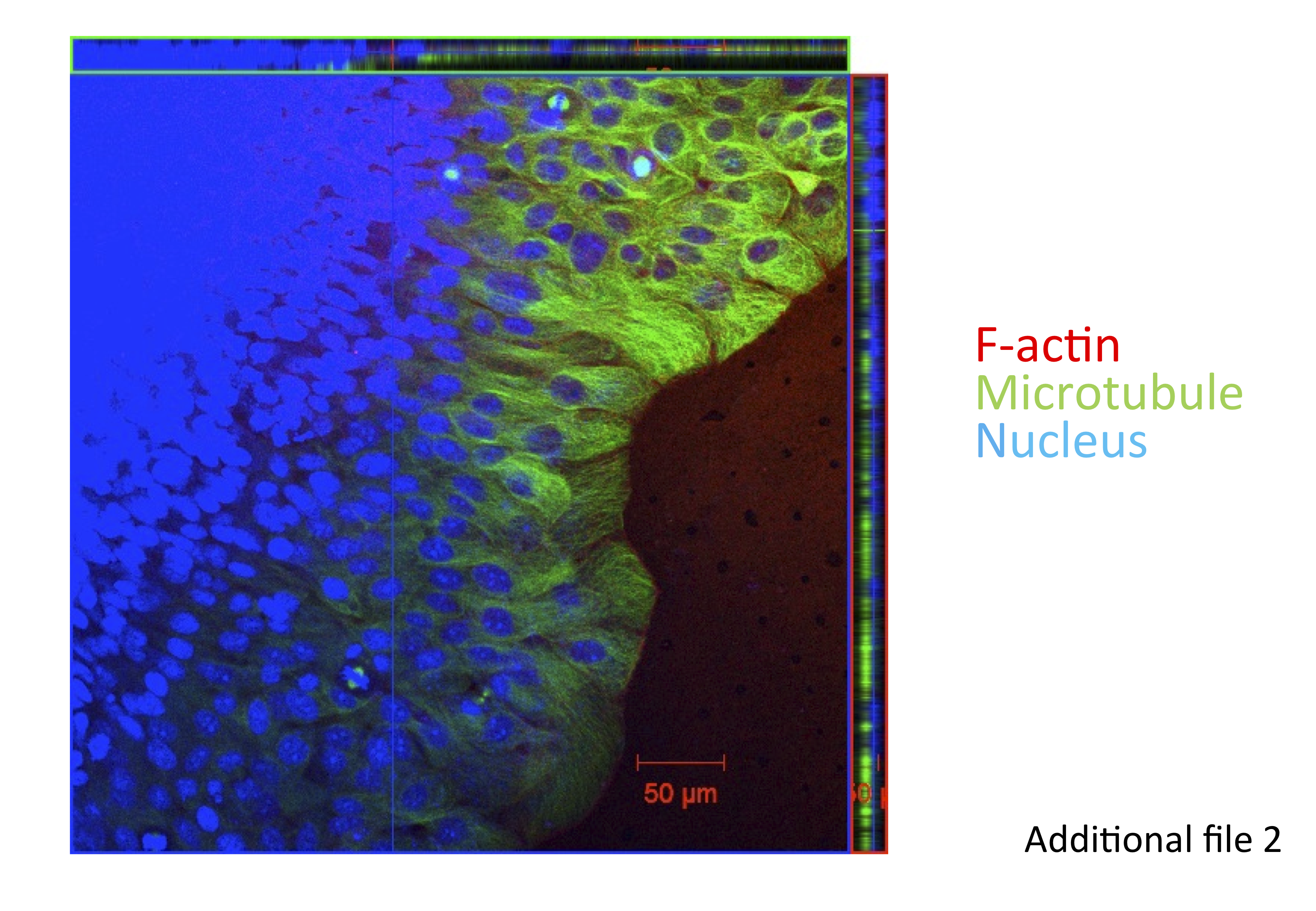

Supplement: Additional file 2. — Reconstructed images of the wounded edge 72 h after scraping. Images were taken with a laser scanning confocal microscope (LSM510; Carl Zeiss, Tokyo, Japan). The cells 5–7 rows behind the wounded edge were observed to be stratified. Red: f-actin, green: microtubules, blue: nuclei. [file 40659_2015_39_MOESM2_ESM.tiff]

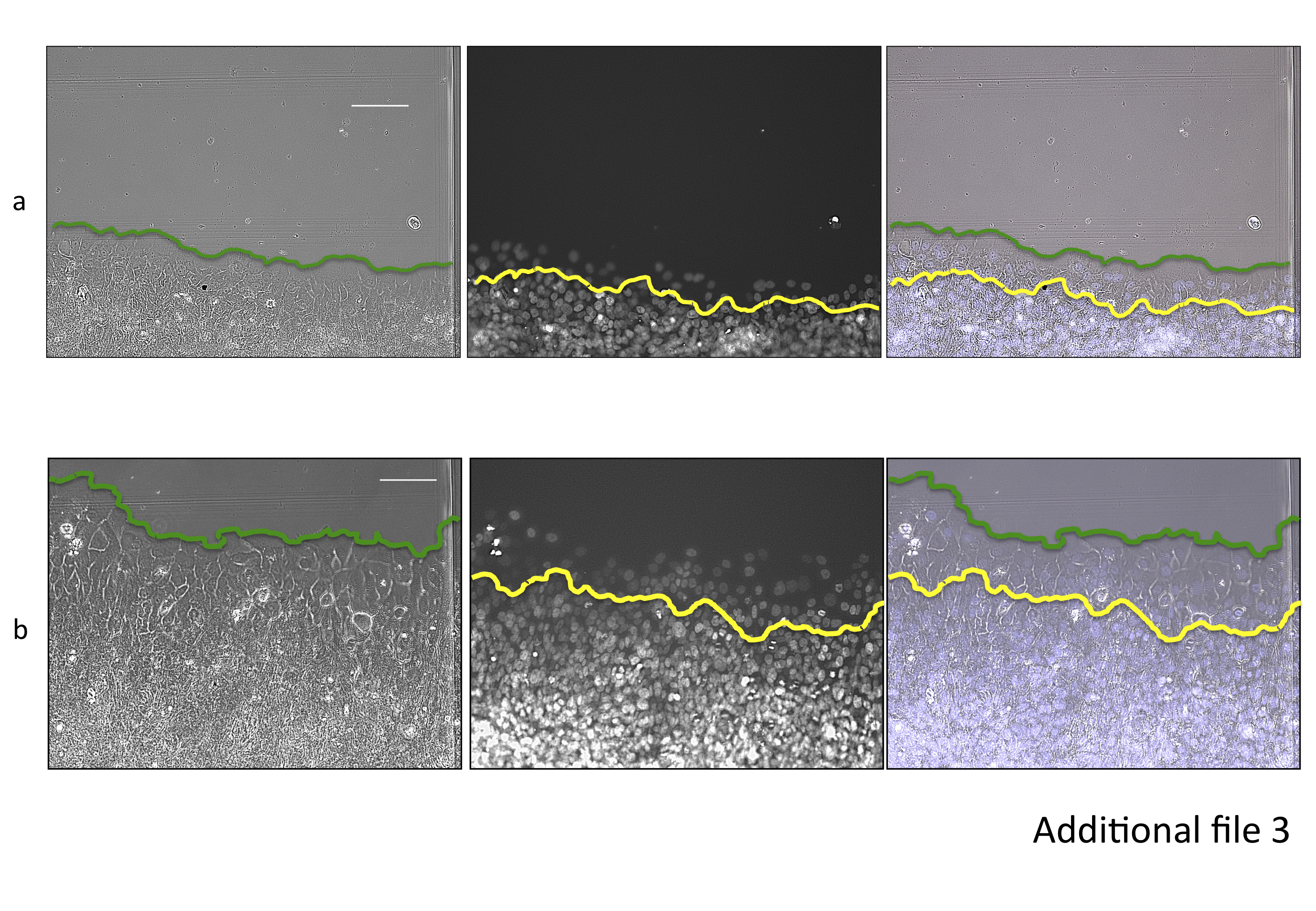

Supplement: Additional file 3. — Images of a randomly selected point along the TE-10 cell sheet. (a) Twenty-four hours after scraping, a simple layer was observed in the first 4–5 rows of the wounded edge, followed by a stratified layer. (b) Seventy-two hours after scraping, the simple layer was observed in the first 4–6 rows of the wounded edge, while the stratified region had also advanced forward over a 48-h period, similar to the wounded edge. As shown in the H2B-GFP nuclear stained images, the simple layer was observed along the wounded edge followed by the stratified layer, similar to the results shown in Fig. 2. Scale bar: 100 µm. Green line: the front line of the cell sheet. Yellow line: the front border of the stratified layer. [file 40659_2015_39_MOESM3_ESM.tiff]

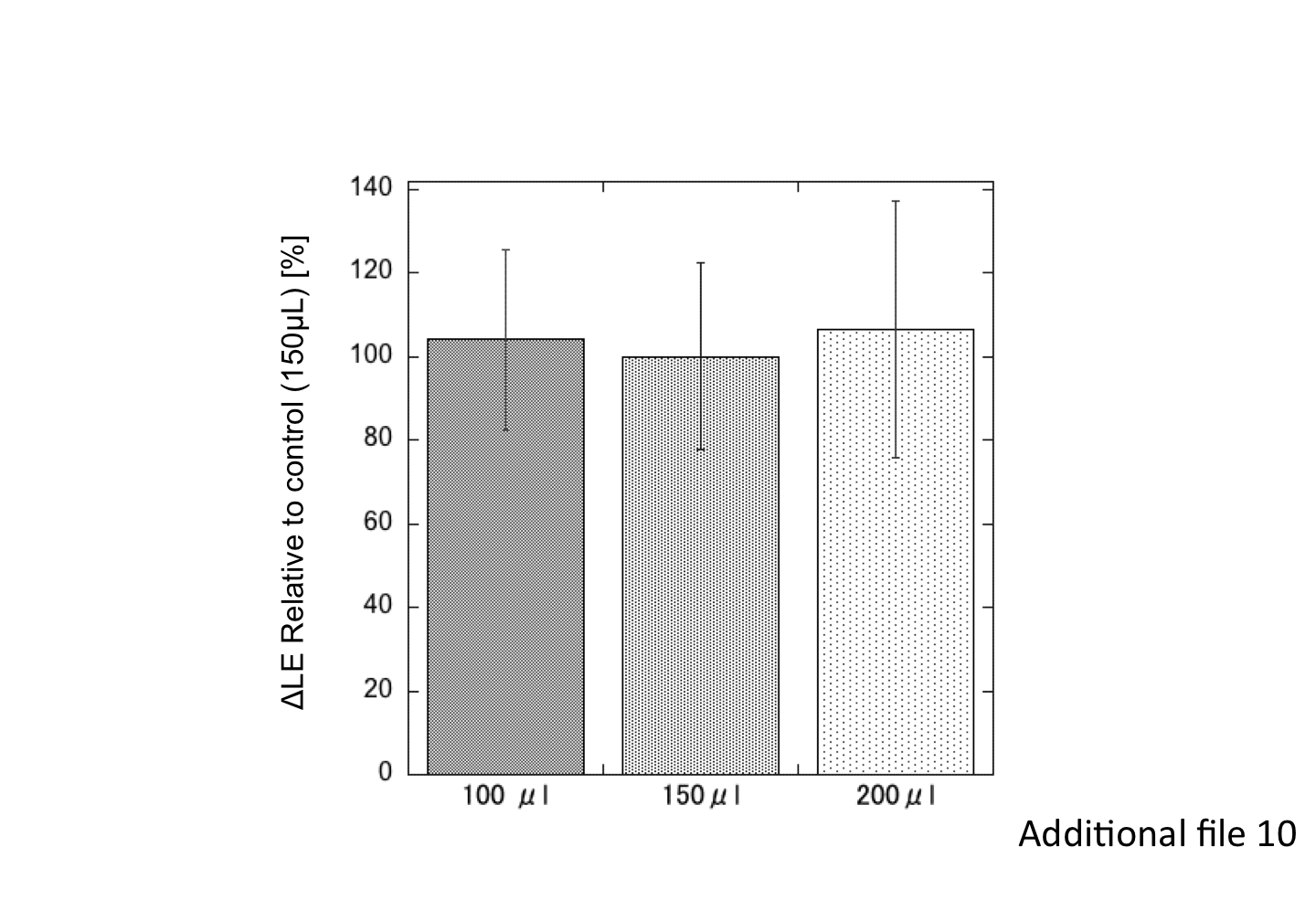

Supplement: Additional file 10. — Relationship between cell numbers and the velocity of the wounded edge. The volume of the cell suspension plated into the hole in the center of each PDMS stencil was increased in 50-µL increments from 100 to 200 µL. Distances migrated during the 48-h period were measured as described in the Materials and Methods section. Experiments were performed in triplicate, and at least 3 samples were studied for each volume plated. Ratios relative to the data for the 150-µL volume are shown as means ± SD. No significant differences were observed. [file 40659_2015_39_MOESM10_ESM.tiff]

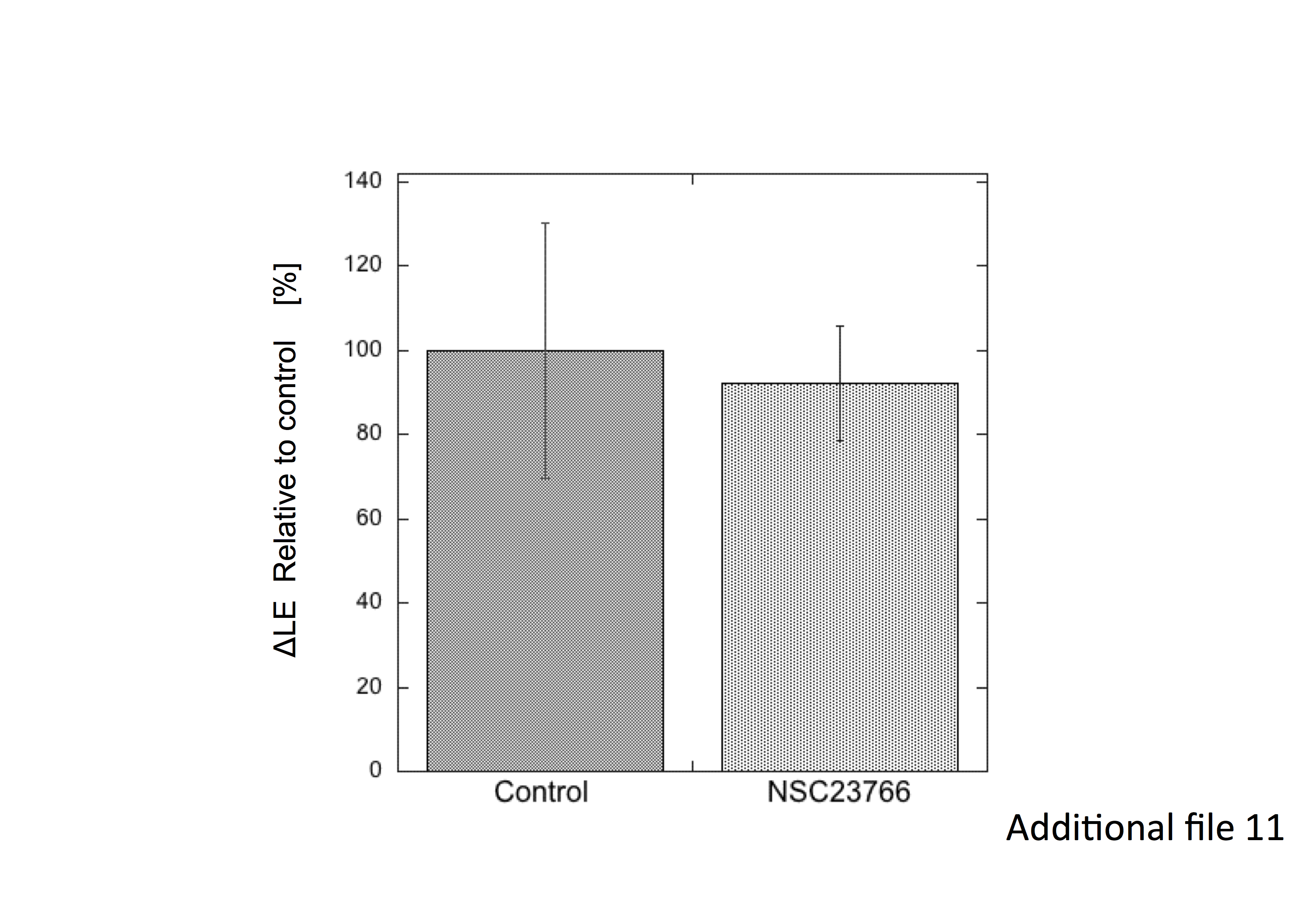

Supplement: Additional file 11. — Effects of a Rac1 inhibitor on the collective migration of TE-10 cells. A series of scrape assays was performed using NSC23766, a well-known inhibitor of Rac1, at a final concentration of 50 µM. Only vehicle was added to the control samples. Data represent the mean ± SEM of the migration distance of the leading edge (ΔLE), using the original data. No significant differences were observed, as determined by a t-test (p = 0.227). Three different samples were prepared for each condition. [file 40659_2015_39_MOESM11_ESM.tiff]

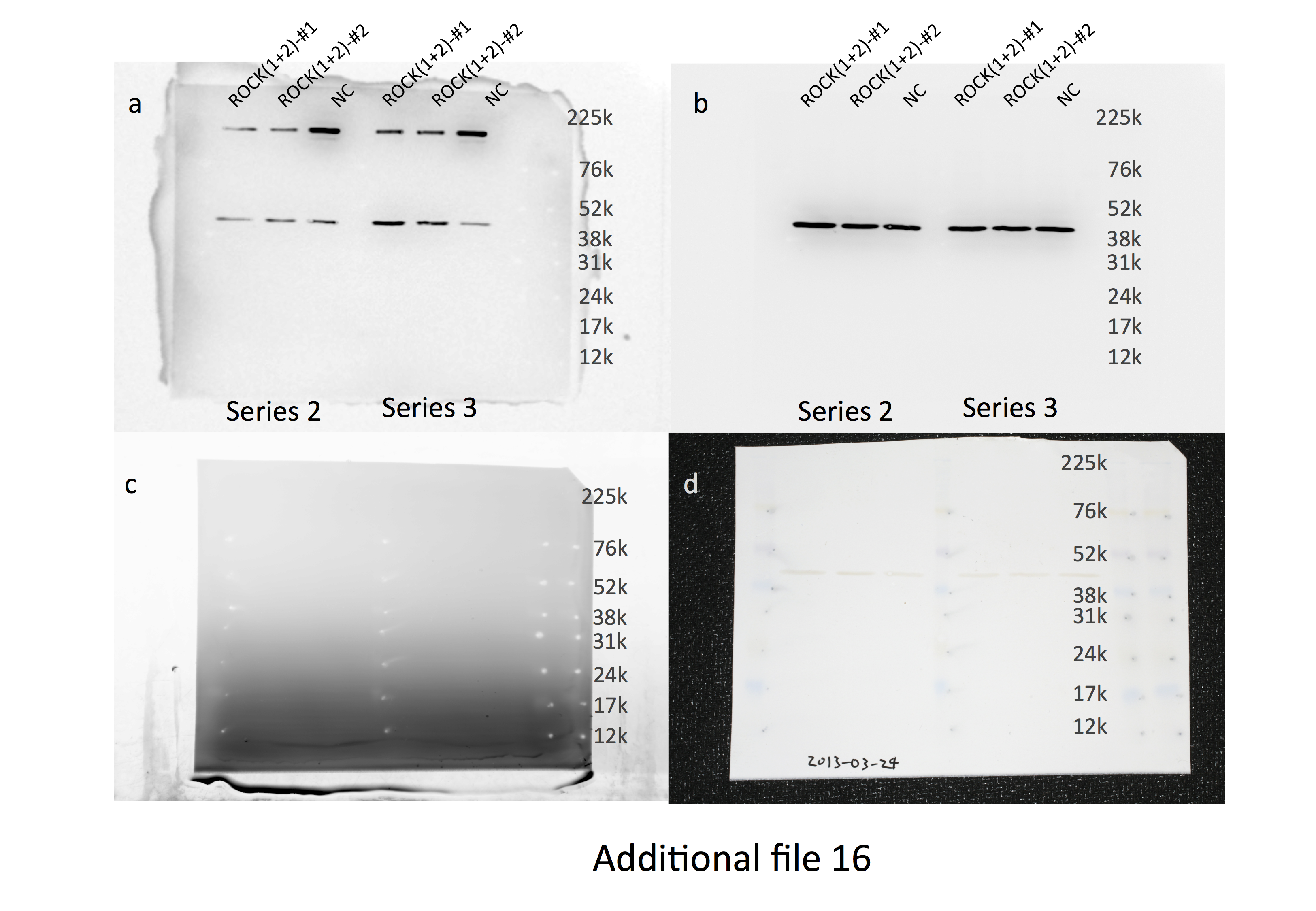

Supplement: Additional file 16. — Original immunoblotting data for ROCK1 and β-actin. (a) The bands of approximately 160 kDa represent ROCK1, while other non-specific bands of approximately 45 kDa are those of β-actin. (b) The bands of approximately 45 kDa represent β-actin. (c, d) The dots on the sheet indicate the positions of each marker. The 150 and 102 kDa markers are obscure, while the yellow, 76 kDa band is relatively clear. The results of the second and the third series of experiments are shown. [file 40659_2015_39_MOESM16_ESM.tiff]

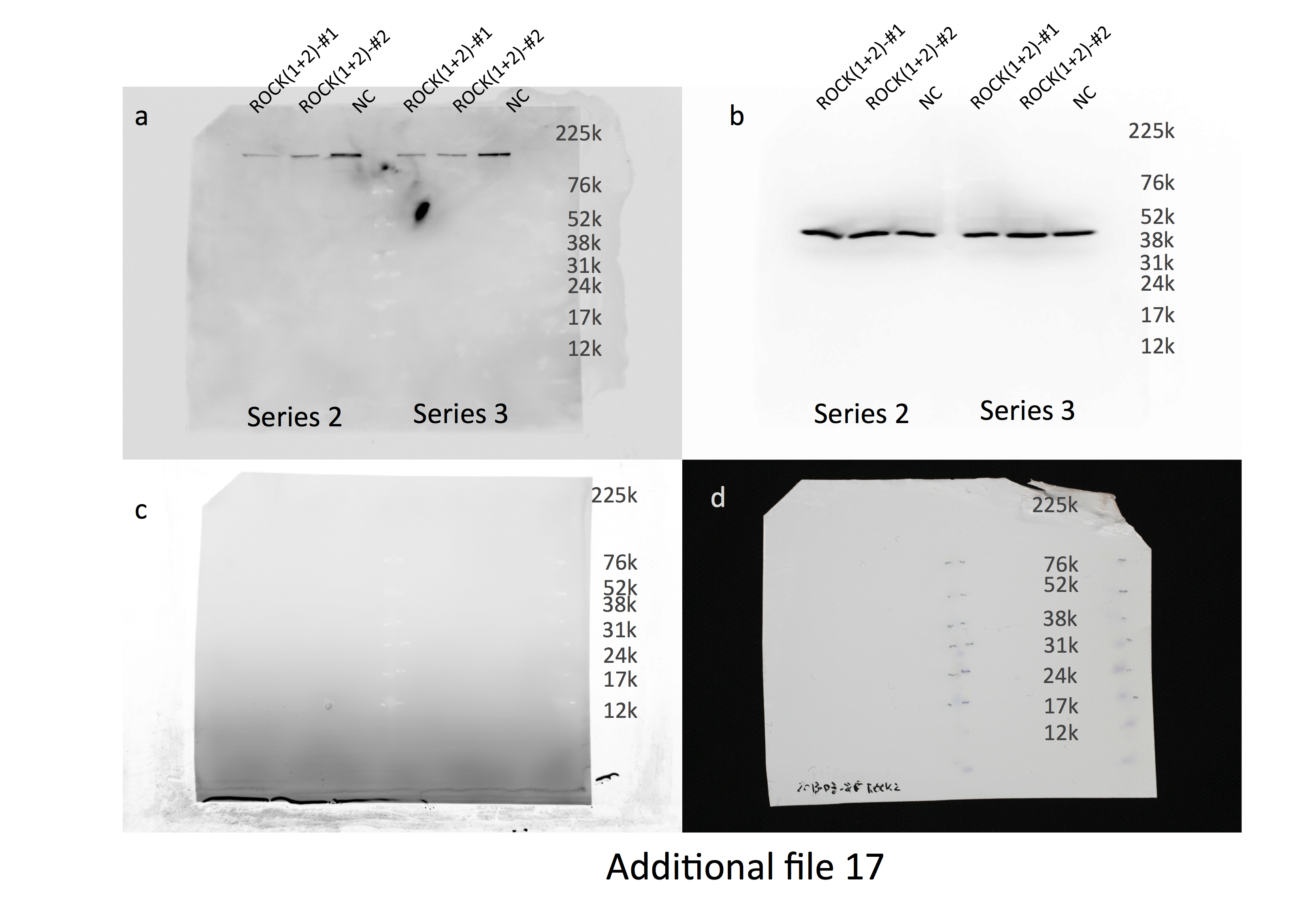

Supplement: Additional file 17. — Original immunoblotting data for ROCK2 and β-actin. (a) The clear bands approximately 160 kDa in size represent ROCK2, while the origin of the band at approximately 52 kDa is unknown. (b) The strong bands of approximately 45 kDa in size originated from β-actin. (c, d) The dots on the sheet show the positions of each marker. The 150 kDa and 102 kDa markers are obscure in the picture, similar to their appearance in Additional file 10. The results of the second and the third series of experiments are shown. [file 40659_2015_39_MOESM17_ESM.tiff]

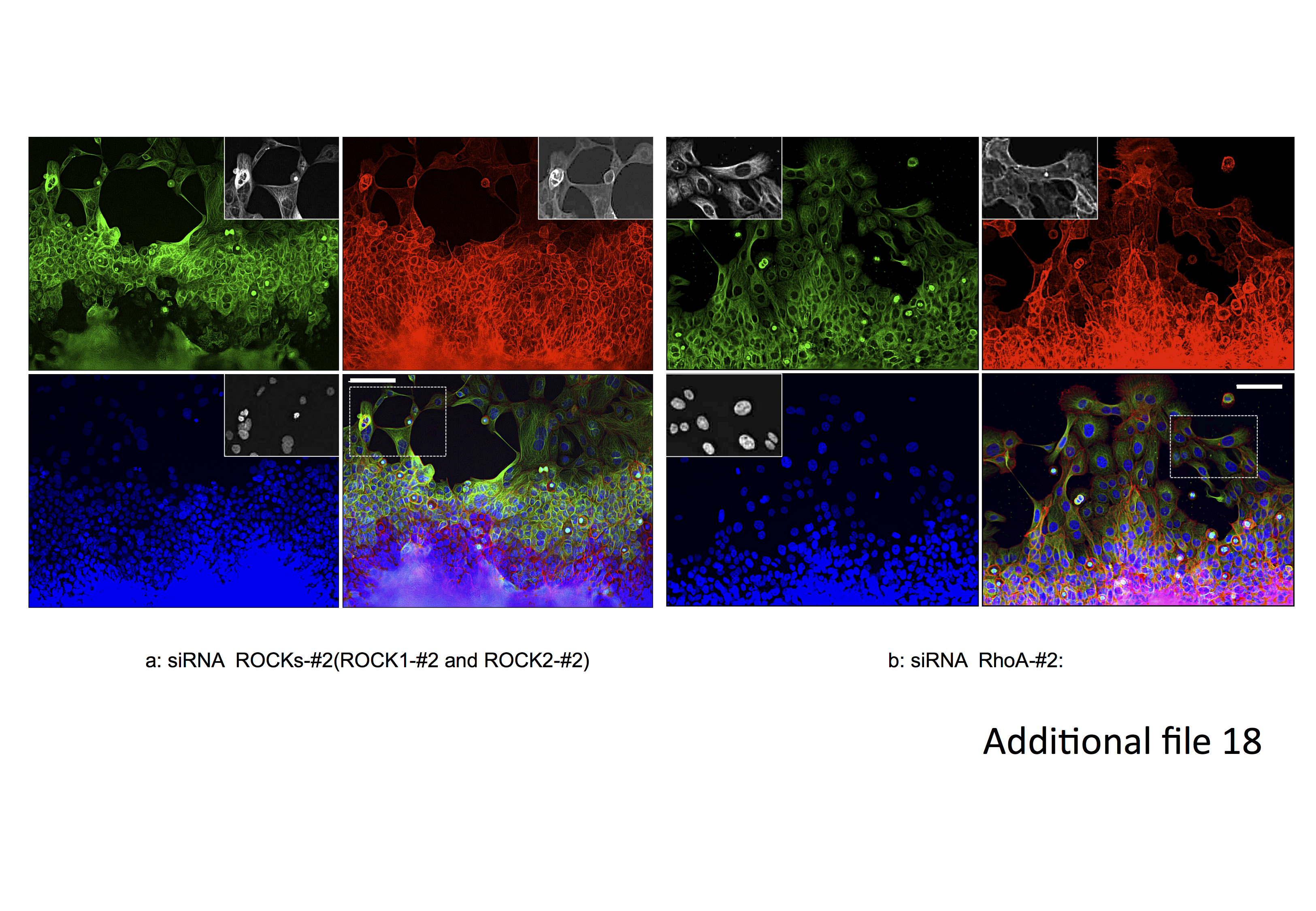

Supplement: Additional file 18. — Images of TE-10 cell sheets with ROCK or RhoA siRNA knockdown 72 h after scraping. (a) TE-10 cells were transfected with siRNAs targeting ROCKs (ROCK1-#2 and ROCK2-#2). The irregular arrangement of the leading row of cells is shown, which was similar to that observed when cells were transfected with ROCK1 siRNA-#1 and ROCK2 siRNA #1. Stress fibers in the leading edge cells were also hypoplastic, although some cells were as large as the negative control cells. (b) TE-10 cells transfected with siRNA #2 targeting RhoA. Spaces between the cells were observed in the simple layer region. The cells in this region were small, with inconspicuous stress fibers, and there were few well-spread, fan-shaped cells. Green: α-tubulin, red: β-actin, blue: nuclei. Each inset shows the magnified image of the area surrounded by a white line on the merged image. Scale bar: 100 µm. [file 40659_2015_39_MOESM18_ESM.tiff]

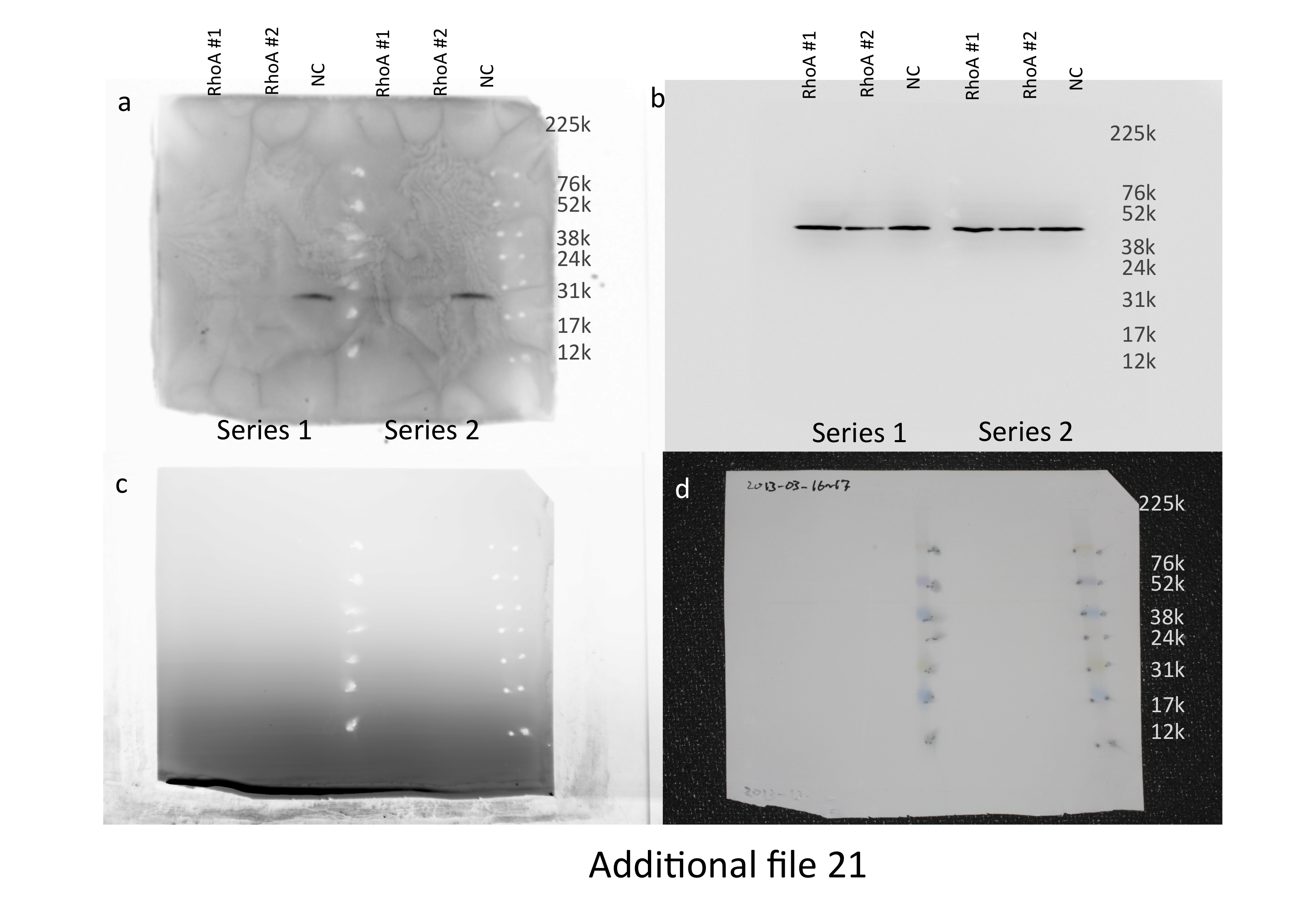

Supplement: Additional file 21. — Original immunoblotting data for RhoA and β-actin. (a) The clear bands of approximately 21 kDa represent RhoA; obscure bands at approximately 21 kDa were observed following RhoA siRNA treatment. (b) The strong bands of approximately 45 kDa in size are β-actin. The white dots reflect the positions of the Rainbow Molecular Weight Markers (GE Healthcare Japan, Tokyo, Japan). (c, d) The dots on the sheet indicate the positions of each marker. The 150 kDa and 102 kDa markers appear obscure, similar to their appearance in Additional file 10. The results of the second and the third series of experiments are shown. [file 40659_2015_39_MOESM21_ESM.tiff]

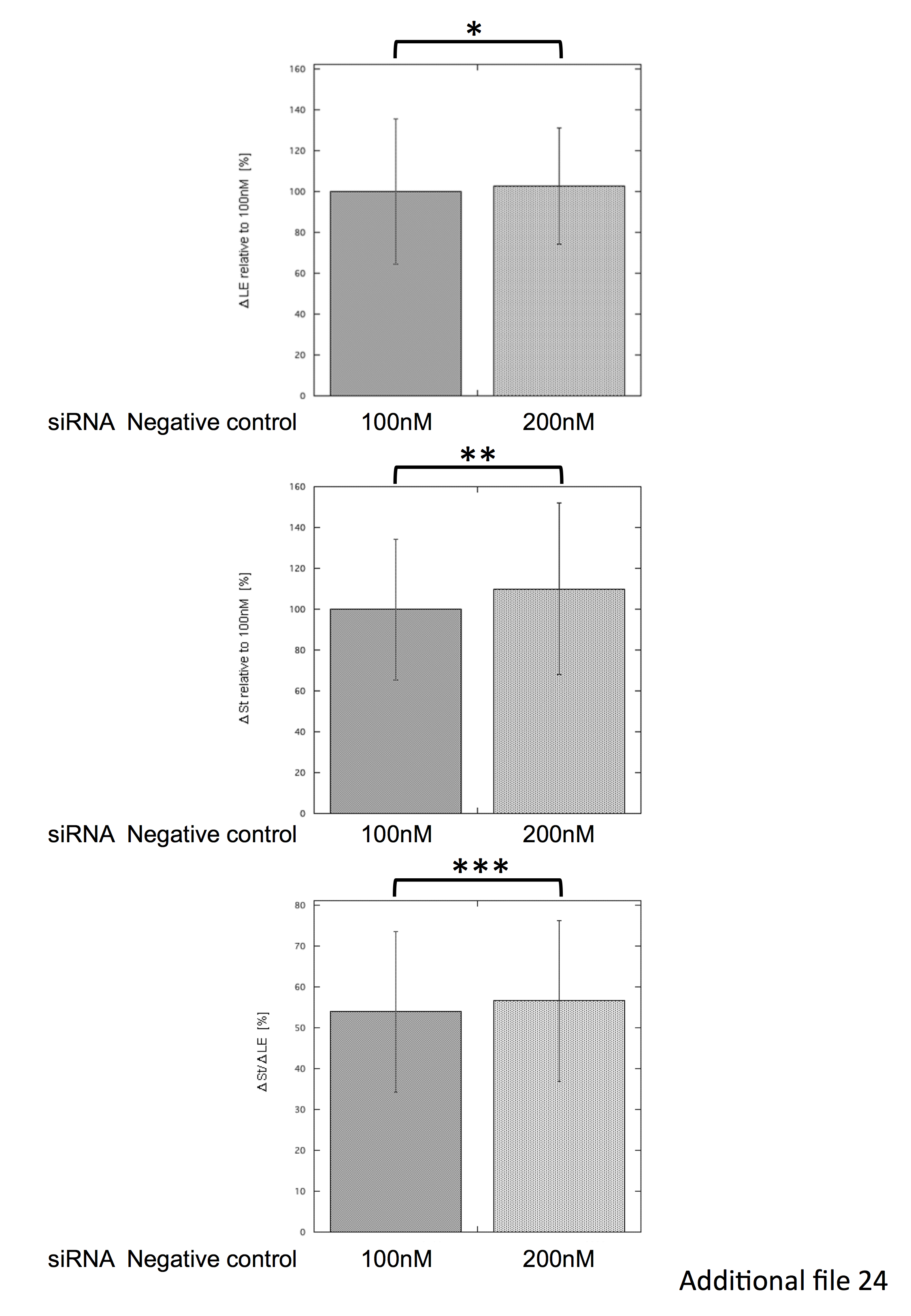

Supplement: Additional file 24. — Viability of TE-10 cells treated with 200 nM siRNA. To investigate the toxicity of high dose siRNA and the viability of the TE-10 cells treated with 200 nM siRNA, ΔLE and ΔSt of 200 nM siRNA treatment of the negative controls were measured and compared with those of control cells treated with 100 nM siRNA. The data of ΔLE and ΔSt of each group were evaluated based on the mean of those of the 100 nM siRNA treated cells, whereas ΔSt/ΔLE were calculated using the original data of each category. The stratified layer of the cells treated with 200 nM siRNA showed slightly faster mobility than the cells treated with 100 nM (** p = 0.005), while there were no significant differences in ΔLE (* p = 0.375) and ΔSt/ΔLE (*** p = 0.136). [file 40659_2015_39_MOESM24_ESM.tiff]
